# Proteomic analysis of RAW macrophages treated with cGAMP or c-di-GMP reveals differentially activated cellular pathways†

**DOI:** 10.1039/c8ra04603d

**Published:** 2018-11-07

**Authors:** Moloud Aflaki Sooreshjani, Ulvi K. Gursoy, Uma K. Aryal, Herman O. Sintim

**Affiliations:** Department of Chemistry, Purdue University West Lafayette IN 47907 USA; Department of Periodontology, Institute of Dentistry, University of Turku Turku Finland; Purdue Proteomics Facility, Bindley Bioscience Center, Purdue University West Lafayette IN 47907 USA; Institute for Drug Discovery and Purdue Institute for Inflammation and Infectious Disease West Lafayette IN 47907 USA hsintim@purdue.edu

## Abstract

Global and quantitative analysis of the proteome help to reveal how host cells sense invading bacteria and respond to bacterial signaling molecules. Here, we performed label free quantitative proteomic analysis of RAW macrophages treated with host-derived cGAMP and bacterial-derived c-di-GMP, in an attempt to identify cellular pathways impacted by these dinucleotides and determine if the host responds differentially to these two cyclic dinucleotides. We identified a total of 3811 proteins of which abundances of 404 proteins in cGAMP and 236 proteins in c-di-GMP treated cells were significantly different compared to the control. Many of the proteins that were strongly and commonly upregulated, such as interferon-induced proteins 47, 202 and 204 (Ifi47, Ifi202, Ifi204), ubiquitin-activating enzyme E7 (Uba7), interferon-induced protein with tetratricopeptide repeats 1, 2 or 3 (Ifit1, Ifit2, Ifit3), ubiquitin-like protein ISG15 (ISG15), might be due to the fact that both dinucleotides promote the production of interferons, which induce the expression of many proteins. However, there were also other proteins that were differentially affected by cGAMP or c-di-GMP treatment, including probable ATP-dependent RNA helicase DHX58 (Dhx58), nuclear autoantigen Sp-100 (Sp100), MARCKS-related protein (Marcksl1) and antigen peptide transporter 2 (Tap2). This is probably due to the differential levels of IFNs produced by the dinucleotides or may indicate that non-STING activation might also contribute to the host's response to c-di-GMP and cGAMP. Interestingly Trex1, a nuclease that degrades DNA (an activator of cGAS to produce cGAMP), was upregulated (3.22 fold) upon cGAMP treatment, hinting at a possible feedback loop to regulate cGAMP synthesis. These results lay a foundation for future studies to better characterize and understand the complex c-di-GMP and cGAMP signaling network.

## Abstract 2

cGAMP modulates proteins involved in antigen presentation and inflammation.

## Graphical abstract

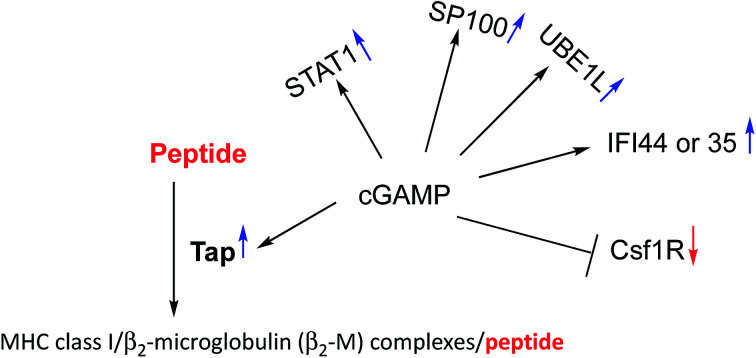

## 1 Introduction

Higher organisms have developed sophisticated mechanisms to sense invading pathogens and mount appropriate responses *via* the innate and adaptive immune systems.^1^ Innate immunity is the early defensive mechanism against pathogens. The innate immune system, composed of dendritic cells, macrophages and neutrophils, utilizes non-specific mechanisms to kill invading pathogens. On the other hand, the adaptive immune system, composed of T and B cells, is specific for each class of pathogens, although cross reactivity can sometimes occur when different pathogens share epitopes.^1,2^ Adaptive immunity leads to a boosted response and immunological memory.^2^ Innate immune cells employ pathogen-recognition receptors (PRR) to sense pathogen-associated molecular patterns (PAMPs). Following detection of PAMPs by PRRs, a series of signaling cascades will be activated, which results in the induction of pro-inflammatory cytokines, such as the transcription factor NF-κB (nuclear factor kappa-light-chain enhancer of activated B cells) and type I interferons (IFNs).^1^ A variety of PAMPs that stimulate the innate immune system, such as LPS, DNA and cyclic dinucleotides, have been documented.^3^

The cGAS/STING pathway, found in various immune, epithelial or endothelial cells, connects the presence of DNA in the cytosol (either host-derived damaged DNA or DNA from invading pathogens) to inflammatory cytokine release. Duplex DNA^4^ or RNA DNA hybrids^5^ in the cytosol are recognized by cyclic GMP-AMP synthase (cGAS), leading to the activation of cGAS to produce cGAMP from ATP and GTP. The host-derived cGAMP then binds to Transmembrane Protein 173 (TMEM173), also called stimulator of interferon genes (STING), which activates TANK-binding kinase 1 (TBK1) to phosphorylate Interferon Regulatory Factor 3 (IRF-3), a main regulator of type I IFN and inflammation response.^6^ Although the activation of the cGAS–cGAMP–STING pathway plays an important protective role against pathogens, persistent activation of this pathway is detrimental and might be the basis of some autoimmune diseases, such as systemic lupus erythematosus.^3,7^

The STING-IRF-3 pathway can also be activated by bacterial-derived cyclic dinucleotides (CDNs). Intracellular bacteria can release cyclic dinucleotides (such as c-di-AMP and c-di-GMP) into the host's cytosol *via* efflux pumps.^8^ Some bacteria cells also autolyze, and this process will release cyclic dinucleotides into the host's cytosol (for intracellular pathogens) or within the host cell's vicinity (extracellular pathogens). C-di-GMP or c-di-AMP activates the STING pathway in a similar fashion to the host-derived cGAMP.^9,10^ Due to the immunostimulatory properties of CDNs, recent efforts have focused on finding compounds that could enhance (potential cancer vaccine adjuvants) or attenuate (anti-inflammatory compounds) the cellular response(s) to cyclic dinucleotides.^3,11^ The complete characterization of how the cell responds to cyclic dinucleotides, including the complete delineation of pathways that are affected by cyclic dinucleotides, would certainly help drug developers in creating compounds to offset the deleterious effects of inflammation due to CDN signaling. Thus far, the activation of the STING-IRF-3 pathway by cyclic dinucleotides has been well characterized, but it is also emerging that cyclic dinucleotides also affect host cells *via* non-STING pathways. In a recent excellent report by Hesketh *et al.*, it was disclosed that when c-di-GMP and c-di-AMP were overexpressed in yeast, several genes were upregulated or downregulated.^12^ Using proteome-wide interaction mapping, Huber *et al.* demonstrated that in addition to STING, cGAMP might bind to other proteins.^13^ Xia *et al.* has reported that c-di-AMP has a higher affinity for endoplasmic reticulum (ER) adaptor, ERAdP, than STING.^14^ McFarland *et al.* also demonstrated that oxidoreductase, aldo-keto reductase family 1, member C13 (AKR1C13) or RECON (reductase controlling NF-κB) binds to c-di-AMP and 3′3′-cGAMP and initiates the activation of NF-κB signaling.^15^ In 2014, one of the authors of this report was part of a team that reported that c-di-GMP could convert immune suppressing myeloid-derived suppressor cell (MDSC) into a phenotype that was immune-stimulating and produced IL-12.^16^ In that same paper, it was also demonstrated that c-di-GMP could activate caspase-3 in murine 4T1 tumor cells, leading to tumor death. From the foregoing, it appears that cyclic dinucleotides might be affecting diverse pathways in various cells in a STING-dependent or STING-independent fashion. However, very little is known about the alternative (STING-independent) pathways that cyclic dinucleotides regulate. We rationalized that since the host-derived cGAMP and bacterial-derived c-di-GMP both bind to STING to activate type I IFN, global proteomics profiling of an immune cell, which has been treated with c-di-GMP or cGAMP, could reveal non-STING pathways that are activated by these metabolites. In other words, substantially differentially expressed proteins and/or pathways would unlikely be related to the activation of the STING pathway because both metabolites activate STING similarly. Alternatively a STING-mediated pathway could account for differences in the level of expressed proteins as the two dinucleotides produce different levels of interferons and cytokines. Herein, this proteomics analysis reveals that although the majority of altered protein levels upon treatment of RAW macrophages could be due to the effects of type I IFN (and hence due to the activation of the STING-IRF-3 pathway by both CDNs), there are many examples whereby the two STING ligands differentially affect the expression levels of some proteins, hinting at possible non-STING pathways that are affected by cyclic dinucleotides.

## 2 Experimental design

### 2.1 Cell stimulation with c-di-GMP or cGAMP

500 000 cells per mL RAW-Blue ISG macrophage cells (InvivoGen) were seeded in 96-well plate. 24 hours later, cells were transfected with c-di-GMP and cGAMP at a final concentration of 100 μM. Reporter activity was quantified after 24 hours by QUANTI-Blue™ kit according to the manufacture's instruction. Briefly, 20 μL of cell supernatant was collected and then added to 200 μL of QUANTI-Blue SEAP detection medium (InvivoGen). Next, the mixture was incubated at 37 °C for 3 hours, and SEAP activity was measured using a Cytation™ 5 Multi-Mode Microplate Reader.

### 2.2 Protein extraction and LC-MS sample preparation

Cells were harvested by centrifugation at 14 000 rpm for 15 min at 4 °C. Harvested cells were gently washed 3× with ice-cold 20 mM Phosphate-Buffered Saline (PBS) buffer, and lysed by homogenization in 8 M urea using Precellys® 24 Bead Mill Homogenizer (Bertin) at 6500 rpm for 2 min. The homogenized cells were centrifuged at 14 000 rpm for 15 min at 4 °C, and the supernatant was transferred to new tubes. Five volume (v/v) of pre-chilled (−20 °C) acetone was added to the supernatant and incubated overnight at −20 °C to precipitate the protein. Protein pellets were washed 1× with 80% cold (−20 °C) acetone. After washing, protein pellets were re-dissolved in 8 M urea, and protein concentration was determined by bicinchoninic acid (BCA) assay with BSA as a standard. About 50 μg protein from each sample was reduced (disulfide bonds) by incubating with 10 mM dithiothreitol (DTT) at 55 °C for 45 min, and cysteine alkylated with 20 mM iodoacetamide (IAA) in the dark for 45 min at room temperature. This was followed by incubation with 5 mM DTT again for 20 min at 37 °C to scavenge residual IAA. Proteins were digested using sequencing grade trypsin and Lys-C mix from Promega at a 1 : 25 (w/w) enzyme-to-protein ratio at 37 °C overnight. The digested peptides were cleaned using C18 micro spin columns (The Nest Group Inc.) using the manufacturer's protocol. Peptides were eluted using 80% acetonitrile containing 0.1% formic acid (FA). The samples were vacuum dried and re-suspended in 3% acetonitrile and 0.1% formic acid. The peptide concentration was determined by BCA assay with BSA as a standard. Following BCA, peptide concentration was adjusted to 0.2 μg μL^−1^ and 5 μL was used for LC-MS/MS analysis (see below).

### 2.3 LC-MS/MS data acquisition

Samples were analyzed by reverse-phase HPLC-ESI-MS/MS system using the Dionex UltiMate 3000 RSLC nano System coupled to the Q-Exactive High Field (HF) Hybrid Quadrupole Orbitrap MS and a Nano-electrospray Flex ion source (Thermo Fisher Scientific). Purified peptides were loaded onto a trap column (300 μm ID × 5 mm) packed with 5 μm 100 Å PepMap C18 medium, and then separated on a reverse phase 50 cm long 75 μm ID analytical column packed with 2 μm 100 Å PepMap C18 silica (Thermo Fisher Scientific). For each LC-MS/MS run, 5 μL sample volume containing 1 μg of total peptides was loaded to the trap column and separated using 120 min LC gradient. Mobile phase solvent A was 0.1% formic acid (FA) in water and solvent B was 0.1% FA in 80% acetonitrile. Peptides were loaded to the trap column in 100% buffer A for 5 min at 5 μL min^−1^ flow rate, and eluted with a linear 80 min gradient of 5–30% of buffer B, then changing to 45% of B at 91 min, 100% of B at 93 min at which point the gradient was held for 7 min before reverting back to 95% of A at 100 min. The column was equilibrated at 95% of A for 20 min. Peptides were separated from the analytical column at a flow rate of 300 nl min^−1^. After each 120 min sample run, columns were washed 2× with 30 min linear gradient of 5–45% of B to keep them clean and reduce sample carry over before running the next sample. Column temperature was maintained at 50 °C. The mass spectrometer was operated using standard data-dependent mode. MS data were acquired with a Top20 data-dependent MS/MS scan method. The full scan MS spectra were collected in the 400–1600 *m*/*z* range with a maximum injection time of 100 milliseconds, a resolution of 120 000 at 200 *m*/*z* and AGC target of 3 × 10^6^. Fragmentation of precursor ions was performed by high-energy C-trap dissociation (HCD) with the normalized collision energy of 27 eV. MS/MS scans were acquired at a resolution of 15 000 at *m*/*z* 200 with an ion-target value of 1 × 10^3^ and a maximum injection of 20 milliseconds. The dynamic exclusion was set at 20 s to avoid repeated scanning of identical peptides. Instrument was calibrated every 7 days using calibration mix solution (Thermo Scientific). The performance of the instrument was also evaluated using *E. coli* digest at the start of each batch.

### 2.4 Data analysis

All LC-MS/MS data were analyzed using MaxQuant software (v. 1.6.0.16)^17–19^ with its built-in Andromeda search engine. The MS/MS spectra were searched against the mouse UniProt protein database (downloaded on June 20, 2017) for protein identification and relative quantification. The minimal length of six amino acids was required in the database search. The database search was performed with the precursor mass tolerance set to 10 ppm, MS/MS fragment ion tolerance set to 20 ppm, and enzyme specificity for trypsin and LysC allowing up to two missed cleavages. Oxidation of methionine (M) was defined as a variable modification, and carbamidomethylation of cysteine (C) was defined as a fixed modification for database searches. The ‘unique plus razor peptides’ were used for peptide quantitation. The false discovery rate (FDR) of both peptides and proteins identification was set at 0.01. Proteins labeled either as contaminants or reverse hits were removed from the analysis. Similarly, proteins identified without any quantifiable peak (0 intensity) and those identified by a single MS/MS count were also removed from the analysis. Proteins were clustered based on their elution profiles using hierarchical clustering in Data Analysis and Extension Tool (DAnTE)^20^ and displayed as a heat map. DAnTE was also used to calculate Pearson correlation coefficients of proteins between samples. The heat maps and statistical analyses were performed using Persues and *t*-test analysis. Enrichment and pathway mapping were performed using ingenuity pathways analysis (IPA).

### 2.5 Immunoblotting

Western blot experiments were performed as previously described with slight modification.^21^ RAW macrophage cells were treated with 100 μM cGAMP and 100 μM c-di-GMP. The cells were collected in lysis buffer (20 mM Tris at pH 7.5, 150 mM NaCl, 0.5% NP-40, and 1 mM phenylmethanesulfonyl fluoride). The cell lysates were incubated on ice for 1 hour. Finally, the protein samples were collected by centrifugation at 14 000 rpm for 15 min at 4 °C and separated by SDS-PAGE. The protein samples were transferred to nitrocellulose blotting membrane, and analyzed with specific antibodies against Tap2, SP100, CSF1R, IFI35, IFI44, STAT1, AHNAK2, UBE1L (UBA7) and actin.

Antibodies for Tap2 (A1610), SP100 (A5851), CSF1R (A3019), IFI35 (A16384), IFI44 (A8188) and STAT1 (A12075) were obtained from Abclonal. Antibody for β-actin (3700) was obtained from Cell Signaling Technology. Antibody for UBE1L (TA313018) was obtained from OriGene.

## 3 Results

### 3.1 Cell stimulation with cGAMP and c-di-GMP

**Fig. 1:**
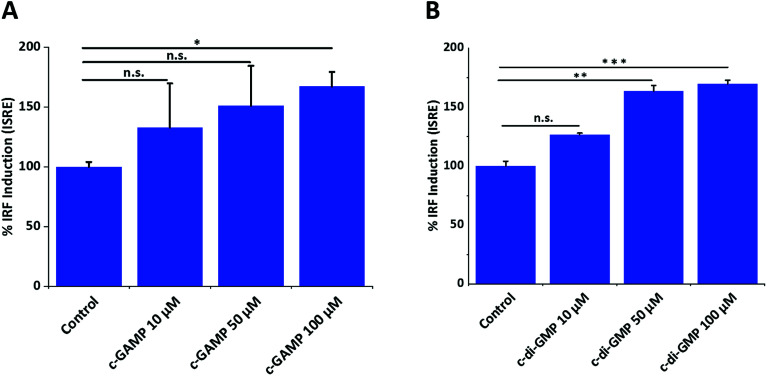
Effects of cGAMP and c-di-GMP on IFN induction. (A) cGAMP (B) c-di-GMP. Error bar is the standard error of three independent experiments. ONE-way ANOVA followed by Dunnett test was performed using GraphPad Prism. Data were plotted using origin.*p value < 0.05, **p value < 0.01, ***p value < 0.001.

To investigate the optimum cyclic-di-nucleotide concentration for IFN-β induction, we treated RAW-Blue ISG macrophage cells with different concentrations of CDN and quantified IFN-β induction (Fig. 1). The concentration of 100 μM was selected for mass spectrometry experiments since it gave a significantly higher IFN signal compared to control.

### 3.2 Global protein identification of RAW macrophage in response to cGAMP and c-di-GMP treatment

We applied MS1 intensity-based label free quantitation (LFQ) to determine changes in protein expression due to cGAMP and c-di-GMP treatments. A prerequisite for LFQ is reproducible peptide intensity measurement in repeated analysis. To measure the variability of LC-MS runs, we analyzed three technical replicates from c-GAMP-treated samples back-to-back. Altogether, we identified 14 280 peptides matching to 3335 proteins in all 3 technical replicates. This represented 81.3% of peptides and 89.8% of proteins commonly identified in all 3 runs (ESI set 1, Fig. S1A and 1B†), suggesting a very good LC-MS reproducibility for protein identification. The average coefficient of variation (CV) of peptides intensities was ∼10% (ESI set 1, Fig. S2, ESI set 2, Table S1†) and *R*^2^ was ∼0.9 (ESI set 1, Fig. S3†), again suggesting high LC-MS reproducibility for protein quantitation. The variability of peptide quantitation was poorly correlated with signal intensities, however, peptides with the highest CV tend to have less signal intensity in agreement with our previous observation.^22^ Other factors such as column quality, column maintenance and calibration of MS instrument, peptide ionization efficiency, or ion suppression, probably also contribute to the variable signals for some of the peptides.^22^ We used a newly acquired Q Exactive Orbitrap HF mass spectrometer for this study, and high sensitivity, resolution and high dynamic ranges of this high end instrument probably underlie the improved accuracy and reproducibility of LFQ quantitation.^23^ One of the major challenges for successful proteomic profiling of multiple samples that vary greatly in protein composition is the accurate alignment of independent LC-MS runs. The freely available MaxQuant software^17–19^ allows to align multiple LC-MS runs for successful quantitative proteomic profiling. Overall, these results showed high reproducibility of our LC-MS platform for protein identification and intensity-based protein quantitation.

### 3.3 Analysis of differentially expressed proteins

**Fig. 2:**
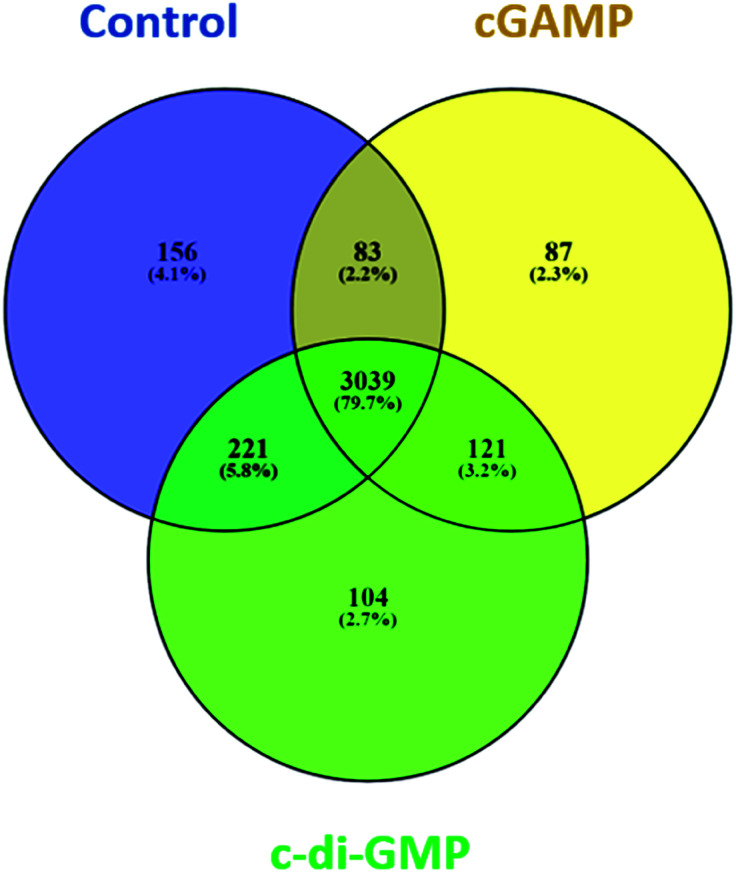
Venn diagram showing the number and the percentage of proteins in each of the three experimental conditions and the overlap of the identified proteins in the three conditions. Data were plotted using the open source Venny software (Venny. 2.1.0).

**Fig. 3:**
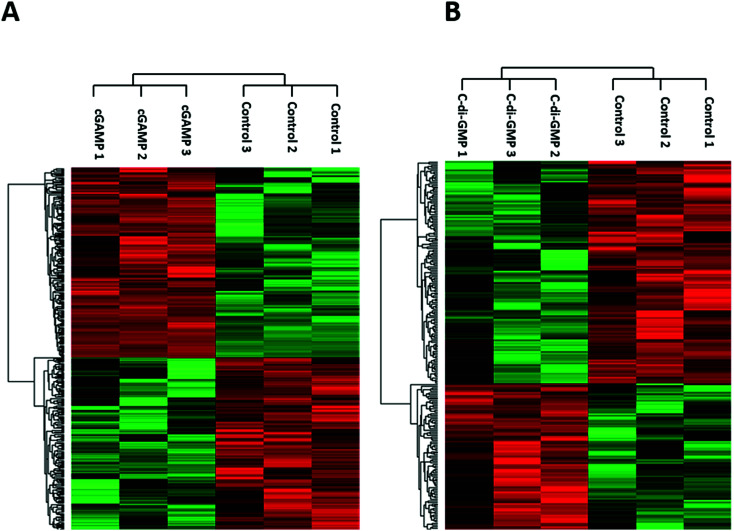
Heatmaps showing hierarchical clustering of differentially expressed proteins due to (A) cGAMP (B) c-di-GMP treatments. LFQ intensities of those significantly different proteins were used for cluster analysis between treated and control samples.

**Fig. 4:**
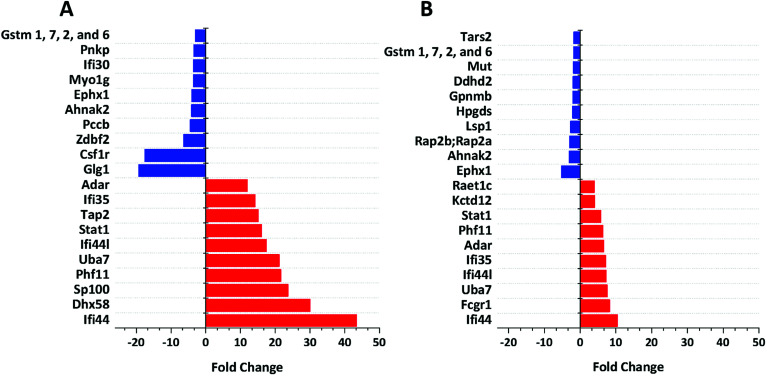
Functional analysis of up- and down-regulated proteins. (A) Top 10 proteins, which are up-regulated (red bars) and down-regulated (blue bars) in response to cGAMP. (B) Top 10 proteins, which are up-regulated (red bars) and down-regulated (blue bars) in response to c-di-GMP. Data were plotted using origin. These were (i) exclusively found in cGAMP treatment: (Lmf1, Prkcd, Uap1, Slc12a7, Capn1, Gpn3, Ddx5, Rpp25l, Ncoa5, Tubgcp3, mKIAA0357, Kdelc2, Eif1ad, C330007P06Rik, Eef1a1, Clec12a, Wwp2, Aim1, H2-T24, M3a,H2-M3, MHC class I, H-2M3, Snapin, Apol9a, Trafd1, Xaf1, H2-T23, Ncapg2, Micu2, Slc39a11, Tmem41b, Ppp2r5e, Tlr3, Pgam2, B2m, A730035I17Rik, Parp3, Gca, Rnf40, Nat2, Tc2n, Camlg, Timm10, Pgm2, D6Wsu163e, Heatr6, Sp1, Chtf18, Phip, Vps33a, Ptpro,PTPphi, Pcdh15, Arfgef3, Tbc1d23, Cecr5, Adck1, Tmem176b, Chrna3, Cwc25, Morf4l2, Asun,Mpa2l, Gbp10, Gbp6, Gbp8, Gbp4, Ogfod1, Stard3, Fam188a, Scyl2, Arhgap27, Crot, Ascc1, Anln, Hspa4l, Rasal2, Dab2ip, Dst, Tacc1, Tacc2, Tbcc,Nptn, Stam2, Mtmr3, Micu1); (ii) exclusively found in c-di-GMP treatment: (Reep5, Stim1, Lpcat4, Itgav, Arih2, Atp2c1, Ddx52, Clec4n, Clec6a, Clcn3, Irf2bp1, Coq9, Uxt, Mmtag2, Samsn1, Srd5a3, Naa35, Emilin2, Ranbp9, Ptgs2, Lpl, Snrpb2, Chid1, Cxcl10, C330027C09Rik;Kiaa1524, Igsf8, Dhps, Psmb10, Rbm34, Pmf1, Spryd7, H2-T23, Ccdc53, COX17, Fam20c, Parp10, Pdcd4, 9230104M06Rik, Crybg3, Bola2, LOC72520, Fam134b, Tmx4, Slc39a11, Sil1, Kif20b, Plau, Haus6;mKIAA1574, Ireb2, Mapk9, Anapc7, Dhx9, Zwint, Trim56, Ccbl2, Acox1, Agpat3, Clpb, Plch1, Mkln1, Rprd1a, Actbl2, Mtm1, Extl2, Abcb11, Mbnl1, Mbnl2, Bicd2, LRWD1, Lrwd1, Kif3a, Fech, Brcc3, Ttn, Exoc1, Sptan1, Hsdl2, Kif21b, Adam15, Tbc1d1, Smek2, Aven, Clptm1l, Itpr3, Thoc3, 10-Sep, Kif13b, Stard9, Kif1c, Kif16b, Kif13a, Naa30, Cd200r1, Las1l, Elovl1, Tgm2); (iii) exclusively found in control group: (Zfp706, gag, Prpf4b, Rras2, Nelfa, Ergic3, Orc5, Xpnpep3, Cx3cr1, Pom121, Abhd6, Ptpn2, Cryzl1, Rnaseh2b, Med22, Ca5b, Kank2, Ppil4, Ankrd44, Guf1, Pkp2, Setd1a, Rtfdc1, Zwilch, D2hgdh, Rsbn1, Rin2, Fam105a, Irf8, Nagk, Rasgrp3, Fam104a, Polr2h, Dph2, Mtpap, Anapc13, Wdr70, Nfatc1, Nubp2, Tbl1x, Sumf1, Cnnm3, Usp9x, L2hgdh, Ankle2, Pus7l, Mon2, Rps6ka4, Nkiras2, 1810009N02Rik, Rgs19, Tti1, Synrg, Lipt2, Mpi,Iscu,Uprt, Rad18, 0610011F06Rik, Arfgap3, Rpusd2, Wbp4, Runx1, Tarbp2, mKIAA0971, Sestd1, Mlycd, Ptpmt1, Hirip3, Znf512, Vps51, Rab3d, Atp6v1g1, Arl15, Rsbn1l, Rpf1, Wdr48, Slc7a5, Arpc5l, Ccs, Plxna2, Akr1b10, Mfsd1, Ccdc88b, Fmr1, Lnp, Slc7a6, Fdx1l, Tbc1d10b, Ranbp10, Klhl9, Napsa, Zfand5, Gcc2, Morc2b, Ints1, Dnase2a, Eif4ebp2, Grcc10, Ccz1, Mtss1, Tfe3, Rab3gap2, Ak1, Tfam, Flad1, Glul, Fam107b, Flcn, Mtfr1l, Maea, Acsf3, Nif3l1, L7rn6, Slc17a5, Snap47, Gemin5, Uqcc1, Mfn2, 5430435G22Rik, Pctk2, Exoc5, Fam206a, Rars2, Irak4, Thtpa, Malt1, Mcat, Adcy7, Rpusd3, Wbscr16, Cdkn1b, Ammecr1l, Tdp1, Ccdc91, Abcc4, uncharacterized protein C19orf52 homolog, Gm21992, Ctu2, Npm3, Ehmt1, Gyg, Wdr74, Pfkm, Mrpl4, Ptpn23, Ddi2, Dph5, Mpc1, uncharacterized protein C4orf3 homolog, Zmynd8, Cbwd1, Eri3, Cerk). Also see ESI set 1, Table S5.†

We identified 3499 proteins in the control, 3330 proteins in the cGAMP treatment and 3484 proteins in the c-di-GMP treatment (ESI set 2, Table S2†), of which 3039 (∼80%) proteins were commonly identified in all the 3 experimental conditions (Fig. 2). Of the 3330 proteins identified in the cGAMP treatment, 404 proteins were significantly different (*p* ≤ 0.05) compared to the control. Of those 404 proteins, 211 proteins were upregulated, and 193 proteins were downregulated in response to cGAMP (ESI set 2, Table S3†). These differentially expressed proteins were clustered into a heatmap as shown in Fig. 3A. The heatmap shows up- and down-regulated proteins, which are distinctly clustered into two groups as compared to the control. In addition, 118 proteins were only detected in the cGAMP treated samples (ESI set 2, Table S4†). The Volcano plot for these up- and down regulated proteins is shown in ESI set 1, Fig. S4A.† The top 10 up- and down regulated proteins in the cGAMP treatment are shown in Fig. 4A. These top 10 up-regulated proteins include interferon-induced protein 44 (Ifi44), probable ATP-dependent RNA helicase DHX58 (Dhx58), nuclear autoantigen Sp-100 (Sp100), PHD finger protein 11 (Phf11), ubiquitin-activating enzyme E7 (Uba7), interferon-induced protein 44-like, minor histocompatibility antigen HA-28 (Ifi44I), signal transducer and activator of transcription (Stat1), antigen peptide transporter 2 (Tap2), interferon-induced 35 kDa protein homolog (Ifi35) and double-stranded RNA-specific adenosine deaminase (Adar). Golgi apparatus protein 1 (Glg1), macrophage colony-stimulating factor 1 receptor (CSF1R), DBF4-type zinc finger-containing protein 2 homolog (Zdbf2), propionyl-CoA carboxylase beta chain, mitochondrial (Pccb), Ahnak2, epoxide hydrolase 1 (Ephx1), unconventional myosin-Ig (Myo1g), gamma-interferon-inducible lysosomal thiol reductase (Ifi30), bifunctional polynucleotide phosphatase/kinase (Pnkp), and glutathione *S*-transferase Mu 1, Mu 7, and Mu 2 (Gstm1, Gstm7, Gstm2, Gstm6x) were the top ten proteins that were down-regulated in the c-GAMP treatment group (Fig. 4A). The full list of proteins that were upregulated or downregulated upon cGAMP treatment can be seen in ESI set 2 (Table S3†).

Similarly, in c-di-GMP treatment, 236 proteins were significantly different from the control group, of which 100 proteins were upregulated, and 136 proteins were downregulated (ESI set 1, Tables S3 and S4, ESI set 2, Table S5†). The heat map of these statistically significant proteins is shown in Fig. 3B. We also identified 103 proteins that were only detected in the c-di-GMP treatment (ESI set 2, Table S6†). The top 10 up- and downregulated proteins are shown in Fig. 4B. The Volcano plot for these up- and downregulated proteins is shown in Fig. S4B.† interferon-induced protein 44 (Ifi44), High affinity immunoglobulin gamma Fc receptor I (Fcgr1), ubiquitin-activating enzyme E7 (Uba7), interferon-induced protein 44-like; minor histocompatibility antigen HA-28 (Ifi44I), interferon-induced 35 kDa protein homolog (Ifi35), the double-stranded RNA-specific adenosine deaminase (Adar), signal transducer and activator of transcription (Stat1), PHD finger protein 11 (Phf11), BTB/POZ domain-containing protein KCTD12 (Kctd12), retinoic acid early-inducible protein 1-gamma (Raet1c) are the top ten proteins that were upregulated in the c-di-GMP-treated group whereas epoxide hydrolase (Ephx1), Ahnak2, Ras-related protein Rap-2b, 2a (Rap2b; Rap2a), lymphocyte-specific protein 1 (Lsp1), hematopoietic prostaglandin D synthase (Hpgds), transmembrane glycoprotein NMB (Gpnmb), phospholipase DDHD2 (Ddhd2), methylmalonyl-CoA mutase (Mut), glutathione *S*-transferase Mu 1,7,2,6 (Gstm1; Gstm7; Gstm2; Gstm6), threonine–tRNA ligase (Tars2) were the top ten proteins that were downregulated in the c-di-GMP treatment group (Fig. 4B). For a full list of proteins that were up- and downregulated upon c-di-GMP treatment, see Table S5 in the ESI† set 2. Many other proteins were also exclusively found in either c-di-GMP or cGAMP treatment groups (but not in the control group), see Table S5 in ESI† set 1.

### 3.4 Functional analysis of differentially expressed proteins

**Fig. 5:**
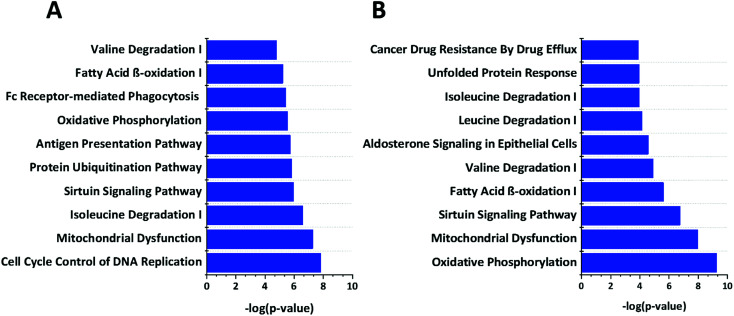
Enrichment analysis of top 10 pathways that are regulated by (A) c-GAMP (B) c-di-GMP. The −log_10_*p*-values below the graph were calculated by IPA software, which represents the magnitude of changes of the whole network. Data were plotted using origin.

We used Ingenuity Pathway Analysis (IPA) based analysis to determine different pathways impacted upon the cGAMP and c-di-GMP treatments. As shown in Fig. 5, cGAMP treatment increased proteins involved with functions in cell cycle control of chromosomal replication, mitochondrial dysfunction, isoleucine degradation, sirtuin signaling pathway, protein ubiquitination pathway, antigen processing pathway, oxidative phosphorylation, fc receptor mediated phagocytosis in macrophage and monocyte, fatty acid β-oxidation and valine degradation in cGAMP treatment (Fig. 5A). Pathway analysis of c-di-GMP treatment indicated an increase in the abundance of proteins related to oxidative phosphorylation, mitochondrial dysfunction, sirtuin signaling pathway, fatty acid β-oxidation, valine degradation, aldosterone signaling in epithelial cells, leucine degradation, isoleucine degradation, unfolded protein response and cancer drug resistance by drug efflux in c-di-GMP treatment (Fig. 5B).

### 3.5 Immunoblotting

**Fig. 6:**
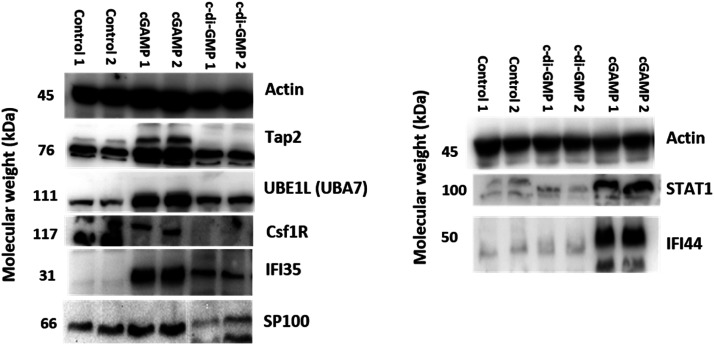
Validation of up and downregulated proteins by western blot analysis. Western blot analysis of Tap2, UBE1L (UBA7), CSF1R, IFI35, SP100, STAT1, and IFI44 in RAW macrophage cells was performed after 24 h treatment with 100 μM cGAMP and 100 μM c-di-GMP. β-actin was used as the control.

We validated our proteomics data by western blot analysis. As observed in Fig. 6, Tap2, UBE1L (UBA7), IFI35, SP100, STAT1, and IFI44 were upregulated after cGAMP treatment, and CSF1R was downregulated after cGAMP treatment. For example, proteomics analysis showed that expression of Tap2 increased by an average of 15.13 fold and CSF1R decreased by an average of 17.55 fold in cGAMP treated RAW macrophages compared to the control (Fig. 4A and ESI set 1, Tables S1 and S2†). As shown in Fig. 6, western blot analysis also showed similar results for these proteins validating the use of label-free quantitative LC-MS/MS analysis for identifying differentially regulated proteins in host cells following cGAMP and c-di-GMP treatments.

## 4 Discussion

Initiating the inflammation response by cyclic dinucleotides has been well-documented, and the identities of binding partners and/or downstream kinases and transcription factors that respond to an intracellular increase in cyclic dinucleotides have been well characterized.^7^ It is now appreciated that the antiviral, antibacterial, as well as, the anticancer (*via* T-cell stimulation) properties of cyclic dinucleotides are derived from the ultimate production of type I IFN.^24^ However, a system-wide characterization of how different cyclic dinucleotides might affect different pathways has not been carried out. To unravel this complexity, we have performed MS-based quantitative proteomics and characterized protein levels and pathways, which significantly changed upon cyclic dinucleotide addition. Others have partially addressed this issue. For example, the excellent work of Hesketh and co-workers revealed that several genes in yeast are differentially expressed in the presence of c-di-GMP and c-di-AMP.^12^ However, the work was performed in yeast cells, which might not be an ideal model to identify networks in immune cells that are impacted by CDNs. Huber *et al.* demonstrated that in addition to STING, cGAMP might bind to other proteins in macrophages.^13^ The main goal of the study by Huber *et al.* was to demonstrate that the cellular thermal shift assay (CETSA) can be used to identify protein targets of metabolites or small molecules in cells and cGAMP was used as case study. However, no attempt was made to do a comprehensive mapping of pathways and/or proteins that are affected by cGAMP. In this current work, we have focused on global responses to both host-derived cGAMP and bacterial-derived c-di-GMP in a bid to identify non-STING impacted pathways and also to determine if the host responds differentially to the various cyclic dinucleotides. Our results indicate that both c-di-GMP and cGAMP impact various proteins and pathways that are likely due to their effect on STING signaling, but there are also differences between the responses by these metabolites revealing possible non-STING activations.

### 4.1 Identification of common cGAMP- and c-di-GMP-induced macrophage proteins

Since it has been shown that both cyclic dinucleotides upregulate type I IFN α and β, *via* the STING-TBK1-IRF3 pathway,^24^ it was expected that many of the differentially expressed proteins upon cyclic dinucleotide treatment would be due to the production of the type I IFNs. Interferon receptors on macrophages bind to type I IFNs to promote the upregulation of IL-12, as well as, TNF-α (protective cytokines).^7^ If high levels of type I IFN are sustained, then IL-10 expression is activated, leading to the inhibition of IL-12, TNF-α and IL-1α,β *via* IL-10 suppressive feedback. One, therefore, has to be careful in interpreting global changes in protein levels upon treatment of IFN-promoting molecules, such as cyclic dinucleotides. This is because depending on the strength and duration of the type I IFN that is produced in response to the presence of a particular cyclic dinucleotide, global changes in protein levels could reflect downstream signaling due to IL-12, TNF-α and TNFα and IL-1α,β or lack thereof.

Consistent with the fact that cyclic dinucleotides promote type I IFNs, several proteins that are known to be upregulated upon IFN stimulation were also found to be upregulated upon cell treatment by cGAMP.^25^ For example, interferon-induced protein 44, interferon-induced protein 44-like, minor histocompatibility antigen HA-28, nuclear autoantigen Sp-100, interferon-induced 35 kDa protein homolog, interferon-induced, double-stranded RNA-activated protein kinase, interferon-induced transmembrane protein 3, STAT1, 2-5-oligoadenylate synthase 3 and 2-5-oligoadenylate synthase 1A were upregulated 43.35, 17.43, 23.66, 14.21, 5.92, 3.34, 16.06, 5.45, and 4.34 fold, respectively, upon RAW macrophage treatment with cGAMP (ESI set 1, Table S1†). Some of these protein classes have also been shown by others to be upregulated when host cells were treated with other pathogen-associated molecular patterns (PAMPs).^26^ For example, Fang *et al.* reported that treatment of fibroblasts with poly(I:C) led to the upregulation of 2-5-oligoadenylate synthases 1 and 2 at 36.9 and 37.7 fold, respectively.^27^ C-di-GMP treatment also led to the upregulation of interferon-induced, double-stranded RNA-activated protein kinase (3.71 fold) and interferon-induced transmembrane protein 3 (2.01 fold) but did not significantly affect colony-stimulating factor-1 receptor (CSF1R), which was downregulated upon cGAMP treatment. These differences could be due to the various levels of type I IFNs that were produced by cGAMP and c-di-GMP and/or because the two cyclic dinucleotides second messengers bind to different receptors, in addition to STING. Future follow up work, which is beyond the scope of this global proteomics analysis, should help clarify this interesting observation. In addition, gamma-interferon-inducible lysosomal thiol reductase was downregulated 3.53 fold upon cGAMP treatment (ESI set 1, Table S2†).

It has been shown in a few studies that the DNA/cyclic dinucleotides and the dsRNA/RIG-I-MAVS pathways crosstalk and regulate each other.^28^ There are examples of bacterial and RNA viral concomitant infection cases, such as instances when patients with influenza virus infection become secondary infected with *Streptococcus pneumonia* or *vice versa*.^29,30^ In such instances, both the bacterial-derived cyclic dinucleotide and/or host-derived cGAMP that are produced in response to bacterial DNA in the cytosol could augment the levels of type I IFNs and NF-κB, leading to effective suppression of both types of infections. For example, the regulation of RNA infection by cGAS (a DNA sensor that makes cGAMP) has been shown,^31^ but chronic activations of the innate sensing systems could also lead to prolonged inflammation, which is detrimental to the host. Therefore, cross talks that deactivate pathways that lead to inflammation could also be beneficial to the host in the long term. Interestingly, treatment of RAW macrophage with cGAMP led to a massive upregulation (30.04 fold) of probable ATP-dependent RNA helicase DHX58 (also known as LGP2 or Laboratory of Genetics and Physiology 2), which is known to inhibit antiviral signaling through RIG-1.^32^ Could this be an adaptive mechanism whereby the immune system has evolved to limit the damage that would ensue during viral and bacterial co-infection?

The sirtuin pathway is connected to inflammation, but to our knowledge, cyclic dinucleotides have not been shown to regulate the sirtuin pathway. The sirtuins are a family of proteins that mostly have nicotinamide adenine dinucleotide (NAD)-dependent deacetylases activity and play an important role in DNA damage response.^33^ It has been demonstrated that sirtuin 2 (SIRT2) inhibit microglia-mediated inflammation.^34^ In addition, it has been shown that SIRT1 inhibits LPS-stimulated inflammatory pathways in macrophage.^35^ SIRT1 expression is induced by LPS *via* IFN-β mediated activation of the JAK-STAT pathway in macrophages.^36^ Based on these prior reports, we expected that cyclic dinucleotides would also affect the sirtuin pathway since they also induce IFN-β *via* the STING pathway. However, in both the cGAMP and c-di-GMP treated samples, SIRT1 or 2 expressions were not significantly upregulated (using an arbitrary cut off of 1.5 fold change). Instead, our results indicated that c-di-GMP and cGAMP might regulate the sirtuin pathway by modulating the abundance of NADH:ubiquinone dehydrogenase and mitogen activated protein kinase-1. Both cGAMP and c-di-GMP treatments of macrophage led to approximately 1.3 fold (1.48 for cGAMP and 1.31 for c-di-GMP) increase and 1.27 fold decrease in NADH:ubiquinone dehydrogenase and mitogen activated protein kinase-1 levels, respectively.

Immune cells have to adjust metabolism in order to respond to invaders quickly.^37^ The fact that both cGAMP and c-di-GMP treatment increased the levels of key metabolism-related proteins, such as NADH:ubiquinone oxidoreductase (associated with the sirtuin pathway), implied that cellular metabolism would also be impacted. In addition to these aforementioned proteins, both cGAMP and c-di-GMP affected the abundance of most proteins associated with metabolism, such as fatty-acid β-oxidation and oxidative phosphorylation. For example, our results indicated that mitochondrial glutamate carrier 1 (2.72 fold increase) and epoxide hydrolase 1 (3.99 fold decrease), which has a role in the metabolism of lipids, were affected after cGAMP treatment. In addition, cytosolic acyl coenzyme A thioester hydrolase (1.61 fold increase) that is involved in the hydrolysis of acyl-CoA and epoxide hydrolase 1 (5.22 fold decrease) were affected in c-di-GMP treatment. These results are consistent with previous studies that have shown that other immune stimulatory molecules, such as LPS, also upregulate the metabolic pathways.^38^

### 4.2 Identification of expressed proteins in cGAMP treatment

Of the many proteins whose abundance increased in cGAMP but not c-di-GMP treatment, E3 ubiquitin-protein ligase Dtx3L was one of them (DTX3L was upregulated 9.39 fold upon cGAMP treatment of RAW macrophage). DTX3L is a histone E3 ligase, which is involved in DNA damage repair.^39^ Zahng *et al.* has also shown that DTX3L-PARP9 complex promotes ISG expression and thus controls viral infection.^40^ However, it has not been reported that cGAMP can modulate ISG expression through DTX3L-PARP9.

Interestingly, cGAMP affected the abundances (albeit modestly) of cell cycle-related kinases and proteins such as CDK1, CDK2, CDK6, MCM2, MCM3, MCM4, MCM6 and MCM7 by 1.53, 1.50, 1.36, 1.32, 1.25, 1.28, 1.35 and 1.23 fold, respectively. Cyclin-dependent kinases (CDKs) are a family of kinases, which regulate cell cycle progression.^41^ Minichromosome maintenance proteins (MCM) also play important roles in cell replication.^42^ It has been demonstrated that CDKs trigger inflammation by initiating the formation of proinflammatory transcription factors, such as STAT3, NF-κB, AP-1,^43^ so it is interesting that treatment of RAW macrophage by cGAMP leads to the upregulation of CDK1, 2 and 6 by 1.53, 1.50 and 1.36 fold, respectively. Handschik *et al.* have shown that NF-κB subunit p65 interacted with CDK6 physically and functionally, which results in TNF and chemokine induction.^44^ Although current data support that CDKs are involved in NF-κB pathway signaling, the function of CDKs in IFN response needs to be clarified.

Our results indicated that c-GAMP caused the upregulation of Uba7 (also called ubiquitin-activating enzyme E1-like, UBE1L) by 21.09 fold.^45^ UBE1L mRNA has been shown to be induced after rectinoic acid treatment of acute promyelocytic leukemia (APL), leading to PML/RARα degradation and apoptosis in acute promyelocytic leukemia. It therefore appears that cGAMP treatment of RAW macrophage mirrors the RA treatment of APL.^45^ Further results showed that colony-stimulating factor-1 receptor (CSF1R) expression is downregulated in response to cGAMP by 17.55 fold. CSF1R is a receptor tyrosine kinase that regulates macrophage migration, proliferation and survival.^46^ CSF1R undergoes dimerization followed by autophosphorylation in response to CSF-1 and IL-34.^46^ This leads to a cascade signaling which regulates macrophage function. It has been reported that macrophage populations, raised by CSF-1, are linked to cancer and inflammation.^47^ Some CSF1R inhibitors are currently in clinical trials for cancer therapy.^47^

### 4.3 Identification of differentially expressed proteins in c-di-GMP treated macrophage

Double-stranded RNA-specific adenosine deaminase (ADAR) was also upregulated 7.07 fold upon c-di-GMP treatment. cGAMP treatment also upregulated ADAR by 11.96 fold. ADAR is an interferon-inducible enzyme that converts adenosine to inosine in double stranded RNA to cause destabilization of the dsRNA, which affects sensing of the RNA, thereby affecting the antiviral response.^48^ Since ADAR is an IFN-inducible protein, the fact that it was robustly upregulated by both cGAMP and c-di-GMP is in line with its upregulation by interferon.^49^

Another upregulated protein in the c-di-GMP treatment group was BTB/POZ domain-containing protein, potassium channel tetramerization domain containing 12, KCTD12 (5.79 fold). cGAMP treatment also upregulated KCTD12 by 4.67 fold. The role of KCTD12 in immunity is unclear, but it has been shown to affect the proliferation of other cell types.^50^ Luo *et al.* demonstrated that the over expression of KCTD12 in human uveal melanoma OCM-1 cells inhibited proliferation.^51^ In another report, Li *et al.* showed that colorectal cancer cells stemness was regulated by KCTD12 *via* the ERK pathway.^50^

Retinoic acid early-inducible protein 1-gamma, RAE-1c (4.04 fold) was also upregulated in the c-di-GMP treatment group. Retinoic acid early-inducible protein 1, RAE-1c, is expressed on macrophages in response to pathogenic stimuli.^52^ RAE-1 interacts with NKG2D receptor, found on natural killer (NK) cells, activated macrophages and CD8^+^ T cells. The detection of RAE-1 proteins on macrophages by NK cells provides a mechanism for NK cells to communicate directly with infected macrophages.^53^ RAE-1c was upregulated by both c-di-GMP (4.04 fold) and cGAMP (4.71 fold) treatments. This observation might explain an earlier seminal observation by Lanier and coworkers that infecting macrophages with bacteria or dsDNA virus induced RAE-1 mRNA.^53^ In 2004, when this observation was made, the direct link between dsDNA and cGAMP and/or the link between cGAMP/bacterial-derived c-di-GMP or c-di-AMP and STING pathways were not established. Our results, which shows that both c-di-GMP and cGAMP upregulate RAE-1c by 4.04 and 4.71 fold, respectively, might explain the observation by Lanier *et al.*

MARCKS-related protein: myristoylated alanine-rich C kinase substrate (MARCKS) and MARCKS-related protein (MRP) are found in many cell types and are substrates for protein kinase C, PKC. MRP is involved in cytoskeletal rearrangement and the expression of MRP in macrophages is increased by IFN-γ and TNF-α.^54^ Downregulation of MARCKS-related protein (MRP) occurred in macrophages infected with Leishmania.^54^ Here we show that just as other PAMPs, such as LPS that increase the expression of MRP, c-di-GMP treatment resulted in 3.80 fold increase of MRP whereas cGAMP treatment caused no significant increase in MRP. In this instance, the fact that c-di-GMP causes a higher increase in MRP in macrophages than cGAMP is interesting. If this effect is solely *via* IFN-γ and TNF-α, then the current understanding of how both cyclic dinucleotides activate STING to produce these cytokines does not fully explain this observation.

### 4.4 Validation of proteomics with Western blot

Immunoblotting was used to confirm some of the proteomics data. Consistent with the proteomics data, cGAMP and c-di-GMP administrations generally resulted in the upregulation of UBE1L and IFI35. The immunoblotting experiment also confirmed that cGAMP upregulated UBE1L and IFI35 expressions more than c-di-GMP. From the proteomics experiment cGAMP upregulated the expression of STAT1 (16.06X) and IFI44 (43.35X) more than c-di-GMP (6.32X and 16.84X respectively). Western analyses (Fig. 6) revealed that cGAMP (100 μM) upregulated STAT1 and IFI44 whereas no obvious upregulation was seen upon c-di-GMP administration. STAT1 is involved in cellular responses to interferons. It has been reported that IFN regulatory factor-1 (IRF-1) expression is reduced in Stat1-/-mice.^55^

The proteomics experiment revealed that cGAMP caused 15.13X and 23.66X increase in the levels of Tap2 and Sp100 whereas no significant upregulations were observed with c-di-GMP administration. The immunoblotting confirmed that indeed cGAMP increased Tap2 and Sp100 levels whereas c-di-GMP does not affect the levels of both proteins (compare Fig. 4 and 6, Tables S1 and S3†).

Tap, which belongs to the ATP-binding cassette (ABC) superfamily of transporters, is a heterodimer of TAP1 and TAP2 subunits.^56^ Tap1/2 mediates the translocation of peptides, which are derived from the proteosomal degradation of cytosolic proteins into the ER and both subunits of Tap (1&2) are critical for the translocation of peptides into the ER.^56^ In the ER, the peptides are loaded into MHC class I/β_2_-microglobulin (β_2_-M) complexes, which leave the ER into the Golgi apparatus. In the Golgi apparatus, the complexes undergo maturation before being expressed on the cell surface. Class I molecules interact with NK cells, γδT cells or CD8^+^ CTL. It has been reported that mutation in Tap2 impairs macrophage survival.^57^ IFNα signaling is involved in membrane-associated antigen transport factors (tap) upregulation and immunoproteasome complex.^58^ SP100 is also an inducible IFN protein,^59^ which plays an important role in Papilloma virus infection. It has been shown that Sp100 interacts viral genomes to suppress infectious disease processes.^60^

## 5 Conclusion

It has only been a few years ago when it was demonstrated that host-derived and bacterial-derived cyclic dinucleotides bind to STING to activate cytokine production.^26^ Since then, there has been an explosive growth in research related to cyclic dinucleotide signaling in human cells.^11^ These studies are motivated by the potential utility of drugs that interfere or activate the cGAS/STING axis for the treatment of diverse pathologies. But, do these cyclic dinucleotides also activate other pathways and if so how do these alternative activations enhance or antagonize the STING axis? Our first goal was to experimentally demonstrate that cyclic dinucleotides differentially affect signaling pathways in RAW macrophages, hinting at alternative pathway activations. It is likely that the many of the up/down regulations observed in this study are the indirect effects of the cytokines released *via* STING activation (such as type I IFNS). Many of the proteins that were strongly upregulated were interferon-induced proteins. However, there were many other proteins that were differentially affected by cGAMP or c-di-GMP. Since both metabolites activate STING, albeit at different levels, it is difficult to explain the differential effects on some protein levels. The next goal is to figure out how the differential activations and/or deactivations occur and if these represent bona fide activations of pathways other than the cGAS/STING/IRF3 pathway. Addressing these issues requires substantial resources that are beyond the means or expertise of a single laboratory, and we look forward to others using the insights obtained from this study to advance cyclic dinucleotide research.

## Conflicts of interest

There are no conflicts to declare.

## Supplementary Material

RA-008-C8RA04603D-s001

RA-008-C8RA04603D-s002

## References

[cit1] Iwasaki A., Medzhitov R. (2010). Regulation of adaptive immunity by the innate immune system. Science.

[cit2] Bowdish D. M., Loffredo M. S., Mukhopadhyay S., Mantovani A., Gordon S. (2007). Macrophage receptors implicated in the “adaptive” form of innate immunity. Microbes Infect..

[cit3] Wang M., Sooreshjani M. A., Mikek C., Opoku-Temeng C., Sintim H. O. (2018). Suramin potently inhibits cGAMP synthase, cGAS, in THP1 cells to modulate IFN-beta levels. Future Med. Chem..

[cit4] Li X., Shu C., Yi G., Chaton C. T., Shelton C. L., Diao J., Zuo X., Kao C. C., Herr A. B., Li P. (2013). Cyclic GMP-AMP synthase is activated by double-stranded DNA-induced oligomerization. Immunity.

[cit5] Mankan A. K., Schmidt T., Chauhan D., Goldeck M., Honing K., Gaidt M., Kubarenko A. V., Andreeva L., Hopfner K. P., Hornung V. (2014). Cytosolic RNA:DNA hybrids activate the cGAS-STING axis. EMBO J..

[cit6] Newton K., Dixit V. M. (2012). Signaling in innate immunity and inflammation. Cold Spring Harbor Perspect. Biol..

[cit7] Li T., Chen Z. J. (2018). The cGAS-cGAMP-STING pathway connects DNA damage to inflammation, senescence, and cancer. J. Exp. Med..

[cit8] Woodward J. J., Iavarone A. T., Portnoy D. A. (2010). c-di-AMP secreted by intracellular Listeria monocytogenes activates a host type I interferon response. Science.

[cit9] Kalia D., Merey G., Nakayama S., Zheng Y., Zhou J., Luo Y., Guo M., Roembke B. T., Sintim H. O. (2013). Nucleotide, c-di-GMP, c-di-AMP, cGMP, cAMP, (p)ppGpp signaling in bacteria and implications in pathogenesis. Chem. Soc. Rev..

[cit10] Ouyang S., Song X., Wang Y., Ru H., Shaw N., Jiang Y., Niu F., Zhu Y., Qiu W., Parvatiyar K., Li Y., Zhang R., Cheng G., Liu Z. J. (2012). Structural analysis of the STING adaptor protein reveals a hydrophobic dimer interface and mode of cyclic di-GMP binding. Immunity.

[cit11] Opoku-Temeng C., Zhou J., Zheng Y., Su J., Sintim H. O. (2016). Cyclic dinucleotide (c-di-GMP, c-di-AMP, and cGAMP) signalings have come of age to be inhibited by small molecules. Chem. Commun..

[cit12] Hesketh A., Vergnano M., Wan C., Oliver S. G. (2017). Bacterial Signaling Nucleotides Inhibit Yeast Cell Growth by Impacting Mitochondrial and Other Specifically Eukaryotic Functions. mBio.

[cit13] Huber K. V., Olek K. M., Muller A. C., Tan C. S., Bennett K. L., Colinge J., Superti-Furga G. (2015). Proteome-wide drug and metabolite interaction mapping by thermal-stability profiling. Nat. Methods.

[cit14] Xia P., Wang S., Xiong Z., Zhu X., Ye B., Du Y., Meng S., Qu Y., Liu J., Gao G., Tian Y., Fan Z. (2018). The ER membrane adaptor ERAdP senses the bacterial second messenger c-di-AMP and initiates anti-bacterial immunity. Nat. Immunol..

[cit15] McFarland A. P., Luo S., Ahmed-Qadri F., Zuck M., Thayer E. F., Goo Y. A., Hybiske K., Tong L., Woodward J. J. (2017). Sensing of Bacterial Cyclic Dinucleotides by the Oxidoreductase RECON Promotes NF-kappaB Activation and Shapes a Proinflammatory Antibacterial State. Immunity.

[cit16] Chandra D., Quispe-Tintaya W., Jahangir A., Asafu-Adjei D., Ramos I., Sintim H. O., Zhou J., Hayakawa Y., Karaolis D. K., Gravekamp C. (2014). STING ligand c-di-GMP improves cancer vaccination against metastatic breast cancer. Cancer Immunol. Res..

[cit17] Cox J., Hein M. Y., Luber C. A., Paron I., Nagaraj N., Mann M. (2014). Accurate proteome-wide label-free quantification by delayed normalization and maximal peptide ratio extraction, termed MaxLFQ. Mol. Cell. Proteomics.

[cit18] Cox J., Mann M. (2008). MaxQuant enables high peptide identification rates, individualized p.p.b.-range mass accuracies and proteome-wide protein quantification. Nat. Biotechnol..

[cit19] Cox J., Neuhauser N., Michalski A., Scheltema R. A., Olsen J. V., Mann M. (2011). Andromeda: a peptide search engine integrated into the MaxQuant environment. J. Proteome Res..

[cit20] Polpitiya A. D., Qian W. J., Jaitly N., Petyuk V. A., Adkins J. N., Camp 2nd D. G., Anderson G. A., Smith R. D. (2008). DAnTE: a statistical tool for quantitative analysis of -omics data. Bioinformatics.

[cit21] Xiahou Z., Wang X., Shen J., Zhu X., Xu F., Hu R., Guo D., Li H., Tian Y., Liu Y., Liang H. (2017). NMI and IFP35 serve as proinflammatory DAMPs during cellular infection and injury. Nat. Commun..

[cit22] Aryal U. K., McBride Z., Chen D., Xie J., Szymanski D. B. (2017). Analysis of protein complexes in Arabidopsis leaves using size exclusion chromatography and label-free protein correlation profiling. J. Proteomics.

[cit23] Krey J. F., Wilmarth P. A., Shin J. B., Klimek J., Sherman N. E., Jeffery E. D., Choi D., David L. L., Barr-Gillespie P. G. (2014). Accurate label-free protein quantitation with high- and low-resolution mass spectrometers. J. Proteome Res..

[cit24] Mankan A. K., Muller M., Witte G., Hornung V. (2017). Cyclic Dinucleotides in the Scope of the Mammalian Immune System. Handb. Exp. Pharmacol..

[cit25] Guldner H. H., Szostecki C., Grotzinger T., Will H. (1992). IFN enhance expression of Sp100, an autoantigen in primary biliary cirrhosis. J. Immunol..

[cit26] Danilchanka O., Mekalanos J. J. (2013). Cyclic dinucleotides and the innate immune response. Cell.

[cit27] Fang F., Ooka K., Sun X., Shah R., Bhattacharyya S., Wei J., Varga J. (2013). A synthetic TLR3 ligand mitigates profibrotic fibroblast responses by inducing autocrine IFN signaling. J. Immunol..

[cit28] Liu Y., Lin R., Olagnier D. (2017). RIGulation of STING expression: at the crossroads of viral RNA and DNA sensing pathways. Inflammation Cell Signaling.

[cit29] Madhi S. A., Klugman K. P., Vaccine Trialist G. (2004). A role for Streptococcus pneumoniae in virus-associated pneumonia. Nat. Med..

[cit30] McCullers J. A. (2006). Insights into the interaction between influenza virus and pneumococcus. Clin. Microbiol. Rev..

[cit31] Schoggins J. W., MacDuff D. A., Imanaka N., Gainey M. D., Shrestha B., Eitson J. L., Mar K. B., Richardson R. B., Ratushny A. V., Litvak V., Dabelic R., Manicassamy B., Aitchison J. D., Aderem A., Elliott R. M., Garcia-Sastre A., Racaniello V., Snijder E. J., Yokoyama W. M., Diamond M. S., Virgin H. W., Rice C. M. (2014). Pan-viral specificity of IFN-induced genes reveals new roles for cGAS in innate immunity. Nature.

[cit32] Goubau D., Deddouche S., Reis e Sousa C. (2013). Cytosolic sensing of viruses. Immunity.

[cit33] Sack M. N., Finkel T. (2012). Mitochondrial metabolism, sirtuins, and aging. Cold Spring Harbor Perspect. Biol..

[cit34] Pais T. F., Szego E. M., Marques O., Miller-Fleming L., Antas P., Guerreiro P., de Oliveira R. M., Kasapoglu B., Outeiro T. F. (2013). The NAD-dependent deacetylase sirtuin 2 is a suppressor of microglial activation and brain inflammation. EMBO J..

[cit35] Yoshizaki T., Schenk S., Imamura T., Babendure J. L., Sonoda N., Bae E. J., Oh D. Y., Lu M., Milne J. C., Westphal C., Bandyopadhyay G., Olefsky J. M. (2010). SIRT1 inhibits inflammatory pathways in macrophages and modulates insulin sensitivity. Am. J. Physiol.: Endocrinol. Metab..

[cit36] Yoo C. H., Yeom J. H., Heo J. J., Song E. K., Lee S. I., Han M. K. (2014). Interferon beta protects against lethal endotoxic and septic shock through SIRT1 upregulation. Sci. Rep..

[cit37] Eming S. A., Wynn T. A., Martin P. (2017). Inflammation and metabolism in tissue repair and regeneration. Science.

[cit38] Kamal A. H. M., Fessler M. B., Chowdhury S. M. (2018). Comparative and network-based proteomic analysis of low dose ethanol- and lipopolysaccharide-induced macrophages. PLoS One.

[cit39] Yang C. S., Jividen K., Spencer A., Dworak N., Ni L., Oostdyk L. T., Chatterjee M., Kusmider B., Reon B., Parlak M., Gorbunova V., Abbas T., Jeffery E., Sherman N. E., Paschal B. M. (2017). Ubiquitin Modification by the E3 Ligase/ADP-Ribosyltransferase Dtx3L/Parp9. Mol. Cell.

[cit40] Zhang Y., Mao D., Roswit W. T., Jin X., Patel A. C., Patel D. A., Agapov E., Wang Z., Tidwell R. M., Atkinson J. J., Huang G., McCarthy R., Yu J., Yun N. E., Paessler S., Lawson T. G., Omattage N. S., Brett T. J., Holtzman M. J. (2015). PARP9-DTX3L ubiquitin ligase targets host histone H2BJ and viral 3C protease to enhance interferon signaling and control viral infection. Nat. Immunol..

[cit41] Opoku-Temeng C., Dayal N., Hernandez D. E., Naganna N., Sintim H. O. (2018). Tetrahydro-3H-pyrazolo[4,3-a]phenanthridine-based CDK inhibitor. Chem. Commun..

[cit42] Gou K., Liu J., Feng X., Li H., Yuan Y., Xing C. (2018). Expression of Minichromosome Maintenance Proteins (MCM) and Cancer Prognosis: A meta-analysis. J. Cancer.

[cit43] Schmitz M. L., Kracht M. (2016). Cyclin-Dependent Kinases as Coregulators of Inflammatory Gene Expression. Trends Pharmacol. Sci..

[cit44] Handschick K., Beuerlein K., Jurida L., Bartkuhn M., Muller H., Soelch J., Weber A., Dittrich-Breiholz O., Schneider H., Scharfe M., Jarek M., Stellzig J., Schmitz M. L., Kracht M. (2014). Cyclin-dependent kinase 6 is a chromatin-bound cofactor for NF-kappaB-dependent gene expression. Mol. Cell.

[cit45] Kitareewan S., Pitha-Rowe I., Sekula D., Lowrey C. H., Nemeth M. J., Golub T. R., Freemantle S. J., Dmitrovsky E. (2002). UBE1L is a retinoid target that triggers PML/RARalpha degradation and apoptosis in acute promyelocytic leukemia. Proc. Natl. Acad. Sci. U. S. A..

[cit46] Hume D. A., MacDonald K. P. (2012). Therapeutic applications of macrophage colony-stimulating factor-1 (CSF-1) and antagonists of CSF-1 receptor (CSF-1R) signaling. Blood.

[cit47] Ries C. H., Cannarile M. A., Hoves S., Benz J., Wartha K., Runza V., Rey-Giraud F., Pradel L. P., Feuerhake F., Klaman I., Jones T., Jucknischke U., Scheiblich S., Kaluza K., Gorr I. H., Walz A., Abiraj K., Cassier P. A., Sica A., Gomez-Roca C., de Visser K. E., Italiano A., Le Tourneau C., Delord J. P., Levitsky H., Blay J. Y., Ruttinger D. (2014). Targeting tumor-associated macrophages with anti-CSF-1R antibody reveals a strategy for cancer therapy. Cancer Cell.

[cit48] Nie Y., Hammond G. L., Yang J. H. (2007). Double-stranded RNA deaminase ADAR1 increases host susceptibility to virus infection. J. Virol..

[cit49] Tang Y. D., Na L., Fu L. H., Yang F., Zhu C. H., Tang L., Li Q., Wang J. Y., Li Z., Wang X. F., Li C. Y., Wang X., Zhou J. H. (2015). Double-stranded RNA-specific adenosine deaminase 1 (ADAR1) promotes EIAV replication and infectivity. Virology.

[cit50] Li L., Duan T., Wang X., Zhang R. H., Zhang M., Wang S., Wang F., Wu Y., Huang H., Kang T. (2016). KCTD12 Regulates Colorectal Cancer Cell Stemness through the ERK Pathway. Sci. Rep..

[cit51] Luo L., Cui J., Feng Z., Li Y., Wang M., Cai Y., Wu Y., Jin J. (2017). Lentiviral-mediated overexpression of KCTD12 inhibits the proliferation of human uveal melanoma OCM-1 cells. Oncol. Rep..

[cit52] Pflanz S., Hibbert L., Mattson J., Rosales R., Vaisberg E., Bazan J. F., Phillips J. H., McClanahan T. K., de Waal Malefyt R., Kastelein R. A. (2004). WSX-1 and glycoprotein 130 constitute a signal-transducing receptor for IL-27. J. Immunol..

[cit53] Hamerman J. A., Ogasawara K., Lanier L. L. (2004). Cutting edge: Toll-like receptor signaling in macrophages induces ligands for the NKG2D receptor. J. Immunol..

[cit54] Corradin S., Mauel J., Ransijn A., Sturzinger C., Vergeres G. (1999). Down-regulation of MARCKS-related protein (MRP) in macrophages infected with Leishmania. J. Biol. Chem..

[cit55] Ohmori Y., Hamilton T. A. (2001). Requirement for STAT1 in LPS-induced gene expression in macrophages. J. Leukocyte Biol..

[cit56] Gadola S. D., Moins-Teissserenc H. T., Trowsdale J., Gross W. L., Cerundolo V. (2000). TAP deficiency syndrome. Clin. Exp. Immunol..

[cit57] Lapenna A., Omar I., Berger M. (2017). A novel spontaneous mutation in the TAP2 gene unravels its role in macrophage survival. Immunology.

[cit58] Oguejiofor C. F., Cheng Z., Abudureyimu A., Fouladi-Nashta A. A., Wathes D. C. (2015). Global transcriptomic profiling of bovine endometrial immune response in vitro. I. Effect of lipopolysaccharide on innate immunity. Biol. Reprod..

[cit59] Xu P., Roizman B. (2017). The SP100 component of ND10 enhances accumulation of PML and suppresses replication and the assembly of HSV replication compartments. Proc. Natl. Acad. Sci. U. S. A..

[cit60] Stepp W. H., Stamos J. D., Khurana S., Warburton A., McBride A. A. (2017). Sp100 colocalizes with HPV replication foci and restricts the productive stage of the infectious cycle. PLoS Pathog..

