# Supplementary material for: Proteomic analysis of RAW macrophages treated with cGAMP or c-di-GMP reveals differentially activated cellular pathways
Source: RSC Adv. 2018 Nov 7;8(64):36840–51. doi: 10.1039/c8ra04603d (PMC9089301; doi:10.1039/c8ra04603d)
Supplement: RA-008-C8RA04603D-s002 [file RA-008-C8RA04603D-s002.pdf]

Electronic Supplementary Material (ESI) for RSC Advances.
This journal is © The Royal Society of Chemistry 2018

Proteomic analysis of RAW macrophages treated with cGAMP or c-di-GMP reveals differentially activated cellular pathways

Moloud Aflaki Sooreshjani[1], Ulvi K. Gursoy[2], Uma K. Aryal[3] and Herman O. Sintim[1,2,4*]

[1]Department of Chemistry, Purdue University, West Lafayette, IN47907, USA
[2]Department of Periodontology, Institute of Dentistry, University of Turku, Turku, Finland
[3]Purdue Proteomics Facility, Bindley Bioscience Center, Purdue University, West Lafayette, IN 47907, USA
[4]Institute for Drug Discovery and Purdue Institute for Inflammation and Infectious Disease, West Lafayette, IN47907
Email: hsintim@purdue.edu

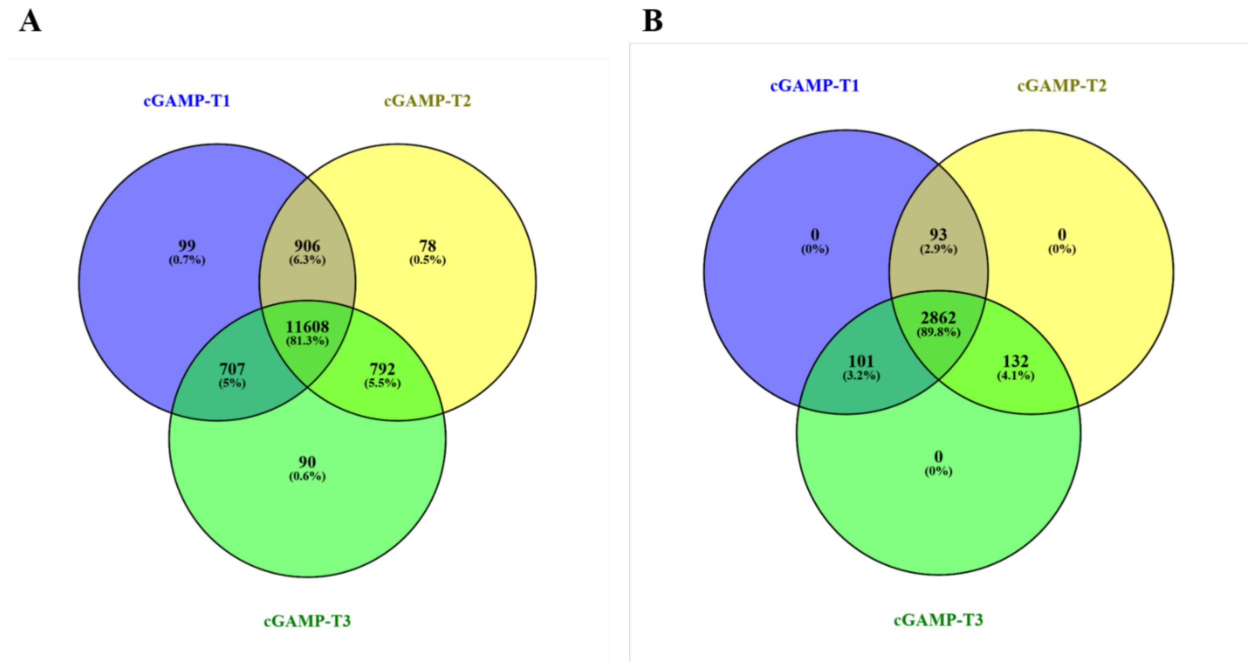

Figure S1. Reproducibility of the LC-MS/MS analysis for peptide and protein identification. (A) Venn diagram showing the overlap of the identified peptides in three technical replicates from cGAMP treated samples. ~81% peptides were commonly identified in all 3 samples. (B) Venn diagram showing the overlap of the identified proteins in three technical replicates from cGAMP treated samples. ~90% proteins were commonly identified in all 3 technical runs. Data were plotted using the open source Venny software (Venny. 2.1.0)

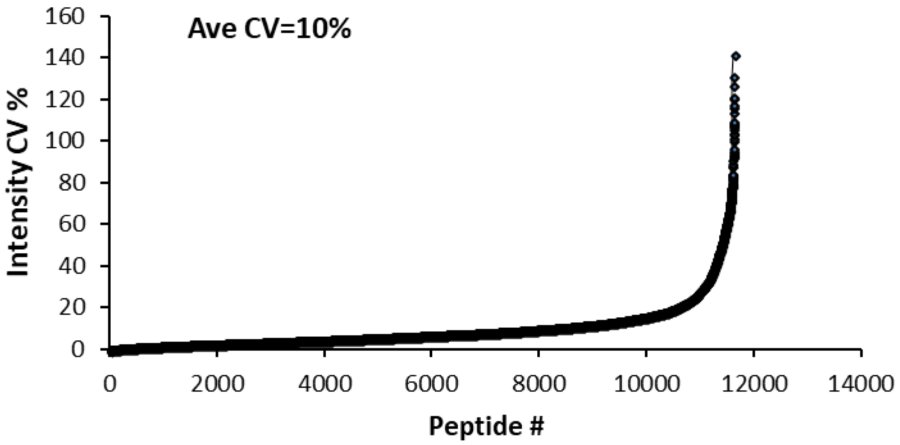

Figure S2. Reproducibility of LC-MS/MS analysis intensity-based for label free quantitation. Coefficient of variation (CV) of MS1 peptide intensity of three technical replicates from cGAMP treated samples

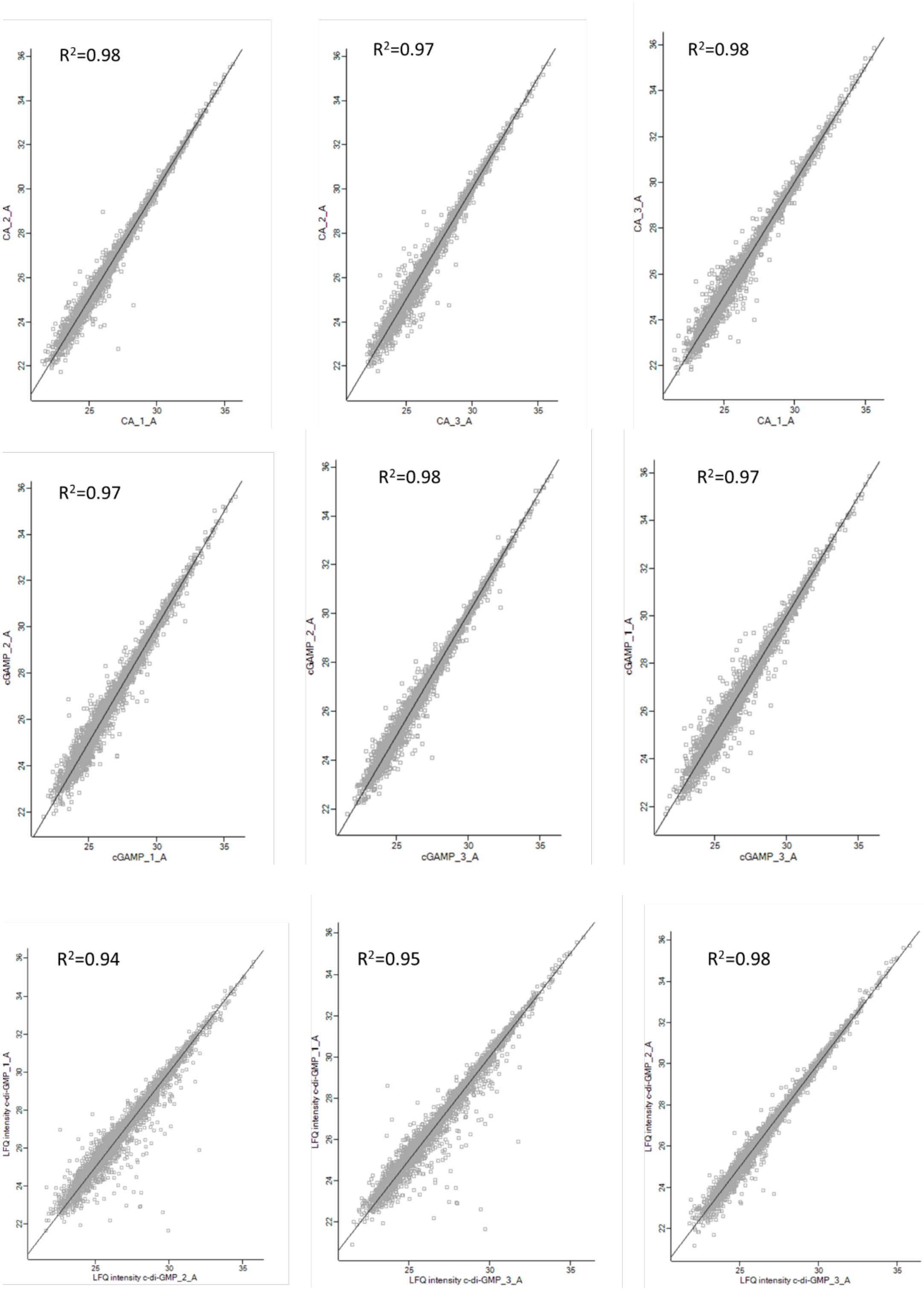

Figure S3. Reproducibility of biological replicates-based protein abundance. Strong correlation was observed among biological replicates.

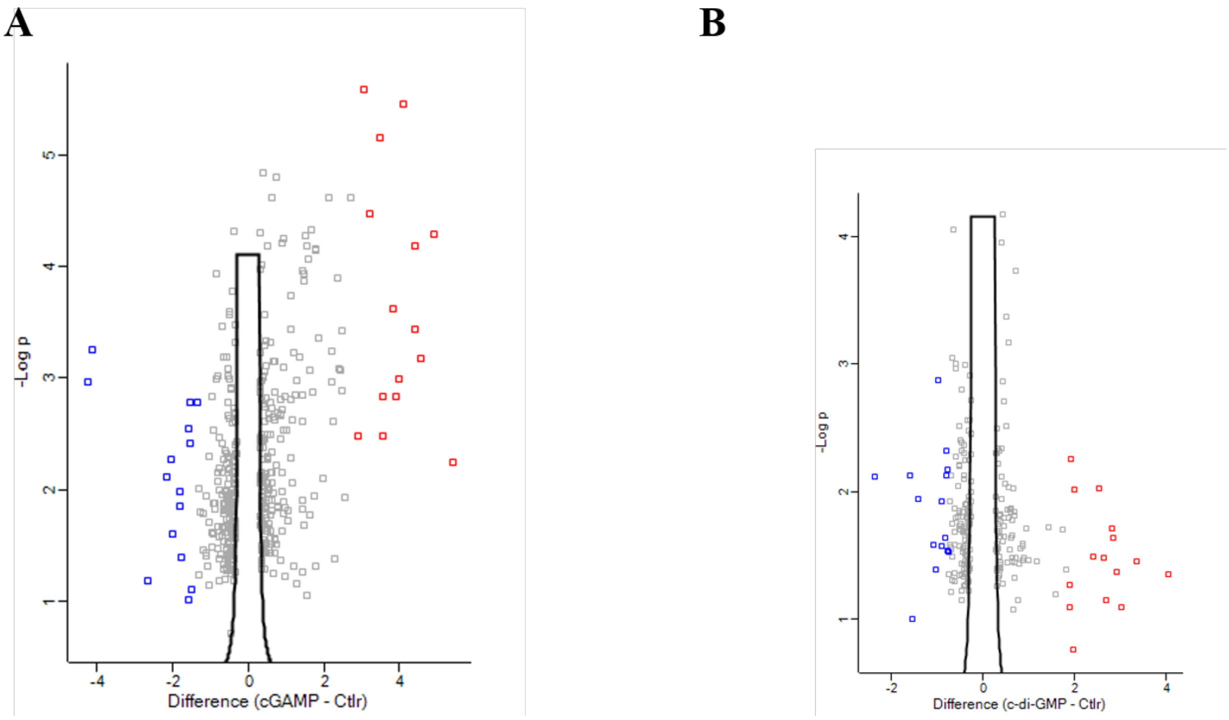

Figure S4. Proteomics Analysis of RAW-ISG cells treated with cGAMP and c-di-GMP. Volcano plot, t test (FDR 0.1, So 0.5) applied to LFQ intensities to determine protein expression pattern in (A) cGAMP treatment. (B) c-di-GMP treatment

Table S1. Top 50 proteins upregulated ($p<0.05$) by cGAMP treatment in Raw ISG macrophages

	Gene names	Description	Fold Change
1	Ifi44	Interferon-induced protein 44	43.35
2	Dhx58	Probable ATP-dependent RNA helicase DHX58	30.04
3	Sp100	Nuclear autoantigen Sp-100	23.66
4	Phf11	PHD finger protein 11	21.57
5	Uba7	Ubiquitin-activating enzyme 7	21.09
6	Ifi44l	Interferon-induced protein 44-like;Minor histocompatibility antigen HA-28	17.43
7	Stat1	Signal transducer and activator of transcription;Signal transducer and activator of transcription 1	16.06
8	Tap2	Antigen peptide transporter 2	15.13
9	Ifi35	Interferon-induced 35 kDa protein homolog	14.21
10	Adar	Double-stranded RNA-specific adenosine deaminase	11.96
11	Ddx58	Probable ATP-dependent RNA helicase DDX58	11.90
12	Mov10	Putative helicase MOV-10	11.09
13	Dtx3l	E3 ubiquitin-protein ligase DTX3L	9.39
14	Rnf213	E3 ubiquitin-protein ligase RNF213	8.28
15	Fcgr1	High affinity immunoglobulin gamma Fc receptor I	7.43
16	Nmi	N-myc-interactor	6.54
17	Eif2ak2	Interferon-induced, double-stranded RNA-activated protein kinase	5.92
18	AI607873		5.64
19	Sp110	Sp110 nuclear body protein	5.60
20	Oas3	2-5-oligoadenylate synthase 3	5.45
21	Parp9	Poly [ADP-ribose] polymerase 9	5.35
22	Irgm1	Immunity-related GTPase family M protein 1	5.12
23	Psmb9	Proteasome subunit beta type;Proteasome subunit beta type-9	4.83
24	Raet1c	Retinoic acid early-inducible protein 1-gamma	4.71
25	Kctd12	BTB/POZ domain-containing protein KCTD12	4.67
26	Lgals3bp	Galectin-3-binding protein	4.58
27	Oas1a;Oas1g	2-5-oligoadenylate synthase 1A	4.34
28	Mlkl	Mixed lineage kinase domain-like protein	3.95
29	H2-L;H2-D1	H-2 class I histocompatibility antigen, L-D alpha chain;H-2 class I histocompatibility antigen, D-B alpha chain	3.68
30	H2-K1;mCG_129835;H2-K	H-2 class I histocompatibility antigen, K-D alpha chain	3.48
31	Pnpt1	Polyribonucleotide nucleotidyltransferase 1, mitochondrial	3.44
32	Tapbp	Tapasin	3.42
33	Ifitm3	Interferon-induced transmembrane protein 3	3.34
34	H2-D1;H2-D;MHC integral membrane protein	H-2 class I histocompatibility antigen, D-D alpha chain	3.30
35	Trex1	Three-prime repair exonuclease 1	3.22
36	Cd180	CD180 antigen	3.07
37	Nt5c3a	Cytosolic 5-nucleotidase 3A	3.06
38	Ly6e	Lymphocyte antigen 6E	3.02
39	Ifi204		2.96
40	Clip1	CAP-Gly domain-containing linker protein 1	2.91
41	Cd47	Leukocyte surface antigen CD47	2.86
42	Pik3ap1	Phosphoinositide 3-kinase adapter protein 1	2.75
43	Samhd1	Deoxynucleoside triphosphate triphosphohydrolase SAMHD1	2.75
44	Icam1	Intercellular adhesion molecule 1	2.75
45	Lcp2	Lymphocyte cytosolic protein 2	2.74
46	Hck	Non-specific protein-tyrosine kinase;Tyrosine-protein kinase HCK	2.73
47	Slc25a22	Mitochondrial glutamate carrier 1	2.72
48	C130026I21Rik		2.71
49	Stxbp1	Syntaxin-binding protein 1	2.68
50	Tor3a	Torsin-3A	2.67

Table S2. Top 50 proteins downregulated (p<0.05) by cGAMP in Raw ISG macrophages

	Gene names	Description	Fold Change
1	Glg1	Golgi apparatus protein 1	19.24
2	Csf1r	Receptor protein-tyrosine kinase;Macrophage colony-stimulating factor 1 receptor	17.55
3	Zdbf2	DBF4-type zinc finger-containing protein 2 homolog	6.38
4	Pccb	Propionyl-CoA carboxylase beta chain, mitochondrial	4.44
5	Ahnak2		4.09
6	Ephx1	Epoxide hydrolase 1	3.99
7	Myo1g	Unconventional myosin-Ig	3.56
8	Ifi30	Gamma-interferon-inducible lysosomal thiol reductase	3.53
9	Pnkp	Bifunctional polynucleotide phosphatase/kinase;Polynucleotide 3-phosphatase;Polynucleotide 5-hydroxyl-kinase	3.43
10	Gstm1;Gstm7;Gstm2;Gstm6	Glutathione S-transferase Mu 1;Glutathione S-transferase Mu 7;Glutathione S-transferase Mu 2;Glutathione S-transferase Mu 6	2.96
11	Mri1	Methylthioribose-1-phosphate isomerase	2.96
12	Atp6v0a1	V-type proton ATPase subunit a;V-type proton ATPase 116 kDa subunit a isoform 1	2.91
13	Lsp1	Lymphocyte-specific protein 1	2.89
14	Xpo7	Exportin-7	2.84
15	Psmb5	Proteasome subunit beta type-5	2.54
16	B4galnt1	Beta-1,4 N-acetylgalactosaminyltransferase 1	2.49
17	Colec12	Collectin-12	2.49
18	Tm9sf2	Transmembrane 9 superfamily member 2	2.39
19	Bphl	Valacyclovir hydrolase	2.29
20	Selenbp1;Selenbp2	Selenium-binding protein 1;Selenium-binding protein 2	2.27
21	Iah1	Isoamyl acetate-hydrolyzing esterase 1 homolog	2.17
22	Sfxn3	Sideroflexin-3	2.17
23	Gpnmb	Transmembrane glycoprotein NMB	2.15
24	Echdc1	Ethylmalonyl-CoA decarboxylase	2.09
25	Msh6	DNA mismatch repair protein Msh6	2.06
26	Sdhc	Succinate dehydrogenase cytochrome b560 subunit, mitochondrial	2.06
27	Acot1;Acot2;Acot6;Acot4	Acyl-coenzyme A thioesterase 1;Acyl-coenzyme A thioesterase 2, mitochondrial;Acyl-coenzyme A thioesterase 6;Acyl-coenzyme A thioesterase 4	2.03
28	Fxyd2	Sodium/potassium-transporting ATPase subunit gamma	1.97
29	Osgep	Probable tRNA N6-adenosine threonylcarbamoyltransferase	1.94
30	Ndufs8	NADH dehydrogenase [ubiquinone] iron-sulfur protein 8, mitochondrial	1.94
31	Hsd17b10	3-hydroxyacyl-CoA dehydrogenase type-2	1.90
32	Pcca	Propionyl-CoA carboxylase alpha chain, mitochondrial	1.89
33	Arhgef6	Rho guanine nucleotide exchange factor 6	1.87
34	Def6	Differentially expressed in FDCP 6	1.85
35	Rcsd1	CapZ-interacting protein	1.84
36	Acyp1	Acylphosphatase;Acylphosphatase-1	1.81
37	Man2b1	Alpha-mannosidase;Lysosomal alpha-mannosidase	1.79
38	Bckdha	2-oxoisovalerate dehydrogenase subunit alpha, mitochondrial	1.75
39	Acy1	Aminoacylase-1	1.75
40	Syngr1	Synaptogyrin-1	1.73
41	Cpt1a	Carnitine O-palmitoyltransferase 1, liver isoform	1.73
42	Syk	Tyrosine-protein kinase;Tyrosine-protein kinase SYK	1.73
43	Serinc3	Serine incorporator 3	1.72
44	Nup35	Nucleoporin NUP53	1.71
45	Tmem205	Transmembrane protein 205	1.71
46	Nagpa	N-acetylglucosamine-1-phosphodiester alpha-N-acetylglucosaminidase	1.71
47	Mrpl49	39S ribosomal protein L49, mitochondrial	1.69
48	Alox5	Arachidonate 5-lipoxygenase	1.69
49	Gtpbp1	GTP-binding protein 1	1.69
50	Ilk	Integrin-linked protein kinase	1.65

Table S3. Top 50 proteins upregulated ($p<0.05$) by c-di-GMP treatment in Raw ISG macrophages

	Gene names	Description	Fold Change
1	Ifi44	Interferon-induced protein 44	16.84
2	Fcgr1	High affinity immunoglobulin gamma Fc receptor I	10.38
3	Uba7	Ubiquitin-activating enzyme 7	8.24
4	Ifi44l	Interferon-induced protein 44-like;Minor histocompatibility antigen HA-28	7.58
5	Ifi35	Interferon-induced 35 kDa protein homolog	7.24
6	Adar	Double-stranded RNA-specific adenosine deaminase	7.07
7	Phf11	PHD finger protein 11	6.53
8	Stat1	Signal transducer and activator of transcription;Signal transducer and activator of transcription 1	6.32
9	Kctd12	BTB/POZ domain-containing protein KCTD12	5.79
10	Raet1c	Retinoic acid early-inducible protein 1-gamma	4.04
11	Hmga1	High mobility group protein HMG-I/HMG-Y	3.93
12	Marcksl1	MARCKS-related protein	3.80
13	Nmi	N-myc-interactor	3.74
14	Eif2ak2	Interferon-induced, double-stranded RNA-activated protein kinase	3.71
15	Lcp2	Lymphocyte cytosolic protein 2	3.54
16	Spp1	Osteopontin	3.38
17	Dtx3l	E3 ubiquitin-protein ligase DTX3L	3.01
18	Dab2	Disabled homolog 2	2.69
19	Yme1l1	ATP-dependent zinc metalloprotease YME1L1	2.27
20	Ifitm3	Interferon-induced transmembrane protein 3	2.01
21	Endod1	Endonuclease domain-containing 1 protein	1.92
22	Dok2	Docking protein 2	1.85
23	Cd44	CD44 antigen	1.83
24	Ehd1	EH domain-containing protein 1	1.83
25	P4ha1	Prolyl 4-hydroxylase subunit alpha-1	1.82
26	Glrx	Glutaredoxin-1	1.78
27	Ttc39b	Tetratricopeptide repeat protein 39B	1.74
28	Syap1	Synapse-associated protein 1	1.70
29	Ero1lb	ERO1-like protein beta	1.65
30	Vapa	Vesicle-associated membrane protein-associated protein A	1.63
31	Hmga2	High mobility group protein HMGI-C	1.63
32	Acot7	Cytosolic acyl coenzyme A thioester hydrolase	1.61
33	Cyb5b	Cytochrome b5 type B	1.61
34	Gaa	Lysosomal alpha-glucosidase	1.60
35	Sec24a	Protein transport protein Sec24A	1.59
36	Katnal2;Spata5	Katanin p60 ATPase-containing subunit A-like 2;Spermatogenesis-associated protein 5	1.58
37	Tes	Testin	1.57
38	Cd47	Leukocyte surface antigen CD47	1.56
39	Pik3ap1	Phosphoinositide 3-kinase adapter protein 1	1.55
40	Mela;gag		1.55
41	Pls3	Plastin-3	1.53
42	Ly6e	Lymphocyte antigen 6E	1.53
43	Creld2	Cysteine-rich with EGF-like domain protein 2	1.52
44	Esd	S-formylglutathione hydrolase	1.50
45	Rnf114	E3 ubiquitin-protein ligase RNF114	1.49
46	gag-pro-pol;gag-pol;mKIAA1466		1.49
47	Rabggta	Geranylgeranyl transferase type-2 subunit alpha	1.48
48	Nbas		1.47
49	Gyk;Gk	Glycerol kinase	1.46
50	Ogfr	Opioid growth factor receptor	1.46

	Gene names	Description	Fold Change
1	Ephx1	Epoxide hydrolase 1	5.22
2	Ahnak2		3.04
3	Rap2b;Rap2a	Ras-related protein Rap-2b;Ras-related protein Rap-2a	2.93
4	Lsp1	Lymphocyte-specific protein 1	2.66
5	Hpgds	Hematopoietic prostaglandin D synthase	2.13
6	Gpnmb	Transmembrane glycoprotein NMB	2.03
7	Ddhd2	Phospholipase DDHD2	1.99
8	Mut	Methylmalonyl-CoA mutase, mitochondrial	1.87
9	Gstm1;Gstm7;Gstm2;Gstm6	Glutathione S-transferase Mu 1;Glutathione S-transferase Mu 7;Glutathione S-transferase Mu 2;Glutathione S-transferase Mu 6	1.86
10	Tars2	Threonine--tRNA ligase, mitochondrial	1.78
11	Psmb5	Proteasome subunit beta type-5	1.75
12	Bphl	Valacyclovir hydrolase	1.74
13	Slc6a11	Transporter;Sodium- and chloride-dependent GABA transporter 3	1.72
14	Pik3r1	Phosphatidylinositol 3-kinase regulatory subunit alpha	1.70
15	Sfxn3	Sideroflexin-3	1.69
16	Bckdha	2-oxoisovalerate dehydrogenase subunit alpha, mitochondrial	1.67
17	Ndufb8	NADH dehydrogenase [ubiquinone] 1 beta subcomplex subunit 8, mitochondrial	1.65
18	Gas7	Growth arrest-specific protein 7	1.65
19	Acot1;Acot2;Acot6;Acot4	Acyl-coenzyme A thioesterase 1;Acyl-coenzyme A thioesterase 2, mitochondrial;Acyl-coenzyme A thioesterase 6;Acyl-coenzyme A thioesterase 4	1.63
20	Ndufb7	NADH dehydrogenase [ubiquinone] 1 beta subcomplex subunit 7	1.63
21	Man2b1	Alpha-mannosidase;Lysosomal alpha-mannosidase	1.63
22	Syngr1	Synaptogyrin-1	1.63
23	Arhgef6	Rho guanine nucleotide exchange factor 6	1.61
24	Serinc3	Serine incorporator 3	1.61
25	Galm	Aldose 1-epimerase	1.60
26	Gnpda1	Glucosamine-6-phosphate isomerase;Glucosamine-6-phosphate isomerase 1	1.59
27	Tpp1	Tripeptidyl-peptidase 1	1.56
28	Vamp4	Vesicle-associated membrane protein 4	1.55
29	Nudt16	U8 snoRNA-decapping enzyme	1.55
30	Dcaf8	DDB1- and CUL4-associated factor 8	1.54
31	Ahnak		1.53
32	Rab11fip5	Rab11 family-interacting protein 5	1.53
33	Celf2	CUGBP Elav-like family member 2	1.51
34	Aldh2	Aldehyde dehydrogenase, mitochondrial	1.51
35	Ndrg3	Protein NDRG3	1.50
36	Rab3il1	Guanine nucleotide exchange factor for Rab-3A	1.50
37	Naglu		1.47
38	Txnrd2	Thioredoxin reductase 2, mitochondrial	1.46
39	Hexa	Beta-hexosaminidase;Beta-hexosaminidase subunit alpha	1.44
40	Git2	ARF GTPase-activating protein GIT2	1.44
41	Uap1l1	UDP-N-acetylhexosamine pyrophosphorylase-like protein 1	1.44
42	Qrsl1	Glutamyl-tRNA(Gln) amidotransferase subunit A, mitochondrial	1.44
43	Add1	Alpha-adducin	1.43
44	Ptms		1.43
45	Gbas	Protein NipSnap homolog 2	1.43
46	Myo5a	Unconventional myosin-Va	1.42
47	Iah1	Isoamyl acetate-hydrolyzing esterase 1 homolog	1.41
48	Pepd	Xaa-Pro dipeptidase	1.40
49	Mdp1	Magnesium-dependent phosphatase 1	1.40
50	Gusb	Beta-glucuronidase	1.39

Table S4. Top 50 proteins downregulated (p<0.05) by c-di-GMP in Raw ISG macrophages<br>Table S5: Proteins in different sections of the Venn Diagram in Figure 2<br>xt).Treatment	Gene Name
**cGAMP**<br>**(Proteins that are exclusively expressed in cGAMP treatment)**	*Lmf1, Prkcd, Uap1, Slc12a7, Capn1, Gpn3, Ddx5, Rpp25l, Ncoa5, Tubgcp3, mKIAA0357, Kdelc2, Eif1ad, C330007P06Rik, Eef1a1, Clec12a, Wwp2, Aim1, H2-T24, M3a, H2-M3, MHC class I, H-2M3, Snapin, Apol9a, Trafd1, Xaf1, H2-T23, Ncapg2, Micu2, Slc39a11, Tmem41b, Ppp2r5e, Tlr3, Pgam2, B2m, A730035I17Rik, Parp3, Gca, Rnf40, Nat2, Tc2n, Camlg, Timm10, Pgm2, D6Wsu163e, Heatr6, Sp1, Chtf18, Phip, Vps33a, Ptpro, PTPphi, Pcdh15, Arfgef3, Tbc1d23, Cecr5, Adck1, Tmem176b, Chrna3, Cwc25, Morf4l2, Asun, Mpa2l, Gbp10, Gbp6, Gbp8, Gbp4, Ogfod1, Stard3, Fam188a, Scyl2, Arhgap27, Crot, Ascc1, Anln, Hspa4l, Rasal2, Dab2ip, Dst, Tacc1, Tacc2, Tbcc, Nptn, Stam2, Mtmr3, Micu1*

**c-di-GMP**<br>**(Proteins that are exclusively expressed in c-di-GMP treatment)**	*Reep5, Stim1, Lpcat4, Itgav, Arih2, Atp2c1, Ddx52, Clec4n, Clec6a, Clcn3, Irf2bp1, Coq9, Uxt, Mmtag2, Samsn1, Srd5a3, Naa35, Emilin2, Ranbp9, Ptgs2, Lpl, Snrpb2, Chid1, Cxcl10, C330027C09Rik;Kiaa 1524, Igsf8, Dhps, Psmb10, Rbm34, Pmf1, Spryd7, H2-T23, Ccdc53, COX17, Fam20c, Parp10, Pdcd4, 9230104M06Rik, Crybg3, Bola2, LOC72520, Fam134b, Tmx4, Slc39a11, Sil1, Kif20b, Plau, Haus6;mKIAA1574, Ireb2, Mapk9, Anapc7, Dhx9, Zwint, Trim56, Ccbl2, Acox1, Agpat3, Clpb, Plch1, Mkln1, Rprd1a, Actbl2, Mtm1, Extl2, Abcb11, Mbnl1, Mbnl2, Bicd2, LRWD1, Lrwd1, Kif3a, Fech, Brcc3, Ttn, Exoc1, Sptan1, Hsdl2, Kif21b, Adam15, Tbc1d1, Smek2, Aven, Clptm1l, Itpr3, Thoc3, 10-Sep, Kif13b, Stard9, Kif1c, Kif16b, Kif13a, Naa30, Cd200r1, Las1l, Elovl1, Tgm2*

**Control**<br>**(Proteins that are exclusively detected in the control)**	*Zfp706, gag, Prpf4b, Rras2, Nelfa, Ergic3, Orc5, Xpnpep3, Cx3cr1, Pom121, Abhd6, Ptpn2, Cryzl1, Rnaseh2b, Med22, Ca5b, Kank2, Ppil4, Ankrd44, Guf1, Pkp2, Setd1a, Rtfdc1, Zwilch, D2hgdh, Rsbn1, Rin2, Fam105a, Irf8, Nagk, Rasgrp3, Fam104a, Polr2h, Dph2, Mtpap, Anapc13, Wdr70, Nfatc1, Nubp2, Tbl1x, Sumf1, Cnnm3, Usp9x, L2hgdh, Ankle2, Pus7l, Mon2, Rps6ka4, Nkiras2, 1810009N02Rik, Rgs19, Tti1, Synrg, Lipt2, Mpi, Iscu, Uprt, Rad18, 0610011F06Rik, Arfgap3, Rpusd2, Wbp4, Runx1, Tarbp2, mKIAA0971, Sestd1, Mlycd, Ptpmt1, Hirip3, Znf512, Vps51, Rab3d, Atp6v1g1, Arl15, Rsbn1l, Rpf1, Wdr48, Slc7a5, Arpc5l, Ccs, Plxna2, Akr1b10, Mfsd1, Ccdc88b, Fmr1, Lnp, Slc7a6, Fdx1l, Tbc1d10b, Ranbp10, Klhl9, Napsa, Zfand5, Gcc2, Morc2b, Ints1, Dnase2a, Eif4ebp2, Grcc10, Ccz1, Mtss1, Tfe3, Rab3gap2, Ak1, Tfam, Flad1, Glul, Fam107b, Flcn,*

	Mtfr1l, Maea, Acsf3, Nif3l1, L7rn6, Slc17a5, Snap47, Gemin5, Uqcc1, Mfn2, 5430435G22Rik, Pctk2, Exoc5, Fam206a, Rars2, Irak4, Thtpa, Malt1, Mcat, Adcy7, Rpusd3, Wbscr16, Cdkn1b, Ammecr1l, Tdp1, Ccdc91, Abcc4, Uncharacterized protein C19orf52 homolog, Gm21992, Ctu2, Npm3, Ehmt1, Gyg, Wdr74, Pfkm, Mrpl4, Ptpn23, Ddi2, Dph5, Mpc1, Uncharacterized protein C4orf3 homolog, Zmynd8, Cbwd1, Eri3, Cerk

**Treatment**	**Gene Name**
**Proteins that are expressed in c-di-GMP and cGAMP treatmentS.**	*Clec4e, Rnf149, Kdm1a, Tdrd7, Kif2a, Il1rn, Erc1;Erc2, Yy1;Zfp42;Yy2, Lrch3, Ascc2, Slfn2, Oxnad1, Gpr84, Secisbp2l, Serpinb9, Cd200r1, Chaf1b, Fndc3a, Nrbp1, Sil1, C5ar1, Hsd17b7, Mospd2, Samd9l, Gbp7, Gm6904;Phf11b, Daxx, Adam17, Gbp4, Oasl2, Ddx3y, Gbp1;Gbp2b, Lst1, NG1;Gpsm3, Dpm1, Apmap, Gbp2, Slfn9, Parp14, Nisch, Isg20, Usp18, Pyhin1;BC094916, Slfn5, Lgals9, Pml, Ass1, Gm5424, Zbp1, Bst2, Ifi47, Ifi202, Ifit2, Siglec1, Isg15, Ifi204, Ifit3, Oasl1, Cmpk2, Fam177a1, Mb21d1, Ifit1, Snx30, Pkn2, Pigk, Vipas39, Stat2, Xpo6, Pex3, Fam134b, Igtp;Ifggd1, Cpne2, Psmg4, Racgap1, Slc12a6;Slc12a4, Esyt2, Znfx1, Agpat3, Srbd1, Mettl10, Scoc, Hsp90ab1, Oas2, Hs1bp3, Ifih1, Strn3, Itm2b, Tbce, Gtf3c4, Imp4, 0610009B22Rik;Trappc2, Ubr2, Raet1, Fam134a, Ccdc6, Aamp, Commd6, Jmjd6, Gss, Eed, Ripk1, Pip4k2a, Ppp2r5c, Llph, Ebf2, Atp6ap2, Rprd1a, Ccdc43, Naa40, Helz2, Fmn1, Rhoc, Iqgap3, Mob4, Slc39a14, Pbk, Itgb5, Irg1*

**Proteins that are expressed in control and c-di-GMP treatment.**	*Trmt10c, Prpf39, Ankmy2, Pole, Ivns1abp, Lsg1, Exosc2, Slc5a3, Gmpr, Ldlrap1, Ikbkg, Bckdhb, Alkbh1, Tmlhe, Cpd, mt-Nd1;ND1;Mtnd1, Scd2, Scd1, Scd3, Akr7a2, Nt5c3b, Aida, Acss2, Sh3pxd2a, Mrpl5, Mrpl11, Hnrnpu, Thoc5, Appl2, Zc3h14, Rnf135, Noa1, Ube2g1, Spg20, Clasp2, Fkbpl, Ifrd2, Ccdc88a, Dido1, Hrsp12, Paxbp1, Psen1, Ints2, Bsdc1, Zdhhc20, Notch2, Engase, Otud7b, Lemd2, Nr3c1, Heatr5a, Cdc42bpb, Sec14l1, Rbm6, Ankrd13a;Ankrd13d, Irak4, Tatdn1, Phkb, Dpcd, Mark3, Mrrf, Nudt16, Mtor, Macf1, Nbas, Kti12, Prcc, Atxn7l3b, Gosr2, 2310035C23Rik;Kiaa1468, Rilpl1, Cog7, Man2c1, Ubr5, Dym, Arhgap18, Papss1, Trmt61a, Qrich1, Pdia5, Rtn4ip1, Myo1d, Lztfl1, Tbc1d2, Coil, Obfc1, Ttc39b, Pign, Ddhd2, Ovca2, Tbk1, Nipsnap3b, Gpr89a, Syvn1, Rbm33, Prps2, Uri1, Tdrkh, Eri1, Pno1, Dnajc1, Mfap1, Zyx, Nubp1, Cd3eap, Oxr1, Pla2g15, Pgap1, Sgpp1, Stx5a;Stx5, Exosc5, Ubr7, Hars2, Tbc1d5, Gamt, Chchd5, Ndufv3, Mta3, Mbp, mKIAA0024;Ptdss1, BC017643, Gopc, Slc38a9, Tmem206, Akap1, Prkar2b, Pogz, Nup43, Cmc1, Scaf4, Paf1, Rad50, Fam3c, Kbtbd11, Mccc2, Afg3l2, Nit1, Mrps18c, Orc3, A430005L14Rik, Mrpl2, Exosc9, Dnmt3a, Smad4, Tmed7, Eny2, Usp50;Polr2l, Toe1, Mettl1, Polr1c, Rbbp5, Yipf3, Cbr3, Fam96b, Echdc1, Galm, Cnot7, Lars2, Eci2, Gstp1, Abcf3, G6pc3, Rbpms, Unc93b1, Cfl2, Wdr6, Tor1aip2, Naga, Wbp2, Psmd10, Usp48, Mrps36, Iws1, Gbas, Polr2d, Cept1, Spg21, Dus3l, Golgb1, Mmgt1, Gaa, Trmt6, Slc23a2, Trove2, Ciao1, Cd63, Mycbp, Zadh2, Aldh1l2, Atp6v0c;Gm15487, Tubg2;Tubg1, Sh3bgrl2, Cwc15, Pcdhga9, Myo7a, Vps36, Hgs, Pop1, Me1, Gng12, Fam63a, Carm1, Ndufa12, Golt1b, Sqrdl, Arl3, Erlin1, Ivd, BC027231, Cars, Ybx1, Ykt6, Ptgs1, Atp6v1d, No66, Apeh, Atp6v1f, Nip7, Katnal2;Spata5, Ints10*
**Proteins that are expressed in control and cGAMP treatment.**	*Chp1, Fabp4, Zfhx3, Timm10b, Cdc27, D2Wsu81e, Ap1g2, Clcn7, Mrpl24, Gpalpp1, Myd88, Suz12, Sntb2, Dnph1, Hint2, Tmem214, Zbtb8os, Armc8, Rela, Spi1, Uvrag, Pacs1, Tk1;Tk1b, Arl8a, Nipsnap1, Fam50a, Kif11, Commd9, Eno3, Asf1b, Fam207a, Pdk3, Rraga;Rragb, Nup88, Chd1, Rasa3, Aarsd1, Gm27029, Emc7, Akap10, Lin7c, Rbbp6, Plcb4, Akr1e2, Vamp7, Sptan1, Vps53, Nfyb, Nr2c2ap, Focad, Lyrm4, Ube3c, 2310022A10Rik, Phc2, Nupl1, Ptp4a2, Pi4k2a, Ints8, Stat5b;Stat5a, Dhx36, Med16, Med24, Pop5, Synrg, Ncbp2, Impact, Irf2bp2, Coa6, Pik3cb, Gins3, Dolpp1, Nsmce1, Commd4, Vps11, Wdr82, Cdc42se1, Mrps21, Chmp3, Cdipt*
